# Plant-Derived Bioactive Compounds: Antioxidation, Autophagy, and Translational Applications in Skin Protection

**DOI:** 10.3390/cimb48040377

**Published:** 2026-04-05

**Authors:** Liangyu Zhu, Mengsha Li, Dianwen Wei, Liping Zhou

**Affiliations:** Department of Natural Products Function, Institute of Natural Resources and Ecology, Heilongjiang Academy of Sciences, Harbin 150040, China; zhuliangyu@haszrs.org.cn (L.Z.);

**Keywords:** plant-derived bioactive compounds, antioxidation, autophagy, bifunctional ingredients, oxidative stress, skin protection mechanisms, translational applications

## Abstract

Oxidative stress from exogenous insults is a major driver of skin aging and hyperpigmentation. Plant-derived bioactive compounds represent promising multifunctional agents with protective effects on skin. They meet the demand for natural, safe skin-protective agents with well-defined action mechanisms. However, current studies lack an integrated understanding of their dual cellular protective mechanisms: antioxidation and autophagy. A unified “component–pathway–efficacy” regulatory network remains lacking, which limits mechanistic insights into skin protection. To address this gap, this comprehensive narrative review retrieved literature from four authoritative databases: PubMed, Web of Science, Scopus, and Wiley Online Library. With targeted keyword retrieval, 129 core studies published between 2021 and 2025 were selected for synthesis. The selection was based on relevance, methodological rigor, and scientific impact. This review constructs a novel “antioxidation–autophagy” synergistic regulatory model. It also establishes a consolidated dual-mechanism framework outlining the “component–pathway–efficacy” axis. This framework reduces knowledge fragmentation across natural product research, skin biology and translational molecular biology. This work integrates the dual protective mechanisms of plant-derived bioactive compounds for skin protection and translational applications. It provides a theoretical basis for understanding their molecular regulatory logic and facilitates further mechanistic studies and translational research on skin protection.

## 1. Introduction

**Figure 1:**
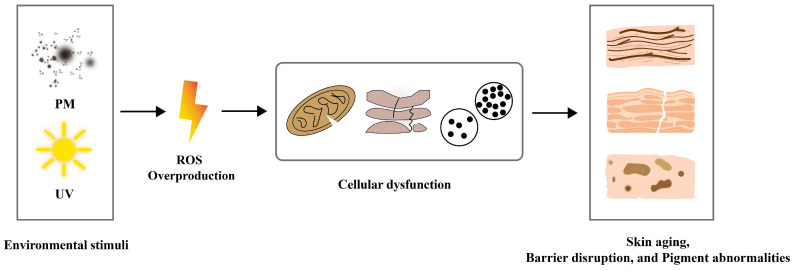
Schematic of skin damage induced by oxidative stress.

The skin, as the body’s largest barrier organ, is constantly exposed to external aggressors such as ultraviolet radiation and environmental pollution, as well as intrinsic metabolic stress [1,2,3]. These factors can trigger oxidative stress, which acts as a key driver of skin aging, hyperpigmentation, and barrier dysfunction (Figure 1). Accordingly, there is a pressing imperative to develop targeted protective strategies that mitigate oxidative stress-mediated skin damage [4,5]. Plant-derived bioactive compounds possess remarkable antioxidant activity and can regulate cellular autophagy. These biological functions contribute to alleviating and repairing skin damage caused by oxidative stress [6,7]. Meanwhile, their natural origin and favorable biocompatibility further make them promising raw materials for the development of skin protective products [8,9]. Euromonitor’s 2024 Beauty Survey shows 21% of global consumers seek plant-derived cosmetic ingredients [10]. Additionally, 40% prioritize natural formulations over cheaper alternatives. The global skincare market is projected to hit $166 billion by 2025 [10]. Such strong consumer demand and broad market prospects place higher requirements on the rational development and application of plant-derived ingredients, further highlighting the importance of understanding their underlying biological mechanisms.

Given the great importance of plant-derived bioactives in skin health, numerous studies have investigated their protective mechanisms. Yet, these studies remain fragmented and lack systematic integration, leaving several key research gaps to be addressed.

In antioxidation mechanisms, plant-derived bioactives modulate classical pathways such as nuclear factor-erythroid 2-related factor 2/antioxidant response element (Nrf2/ARE) and mitogen-activated protein kinase (MAPK), which possess well-established regulatory networks in skin protection [11,12]. Recent studies have focused on refining the regulatory details of these pathways and identifying novel non-canonical antioxidant targets mediated by plant bioactives [13]. These advances provide new avenues for precise regulation of oxidative stress in skin via plant-derived compounds. However, these plant-relevant classical pathways and newly identified non-canonical targets have not been systematically integrated. This insufficient integration hinders the development of rational, precise antioxidant strategies for utilizing plant-derived bioactives in skin protection.

In addition to antioxidant effects, autophagy represents another important mechanism underlying the skin-protective roles of plant-derived bioactives [14]. Early studies primarily investigated the role of canonical autophagy in plant-derived bioactive-mediated skin protection, while current research has shifted its focus to selective autophagy mechanisms [15]. These selective autophagy mechanisms play an important role in alleviating oxidative stress-induced skin damage [16]. However, general autophagy and selective autophagy in the context of plant-derived bioactives have not been systematically integrated or organized into a unified mechanistic framework.

Beyond the independent investigations of antioxidant and autophagy mechanisms, plant-derived bioactives possessing both antioxidant and autophagy-modulating properties have attracted growing attention in multifunctional skincare research. Their “reactive oxygen species (ROS) scavenging + damage clearance” synergy is consistent with precision skincare needs [17,18]. However, over the past five years, there has been no unified framework to synthesize the characteristics of these compounds. Furthermore, the summary of their core synergistic mechanism networks for multifunctional skincare applications remains insufficiently systematic.

Mechanistic studies of plant-derived bioactives in antioxidant and autophagic regulation have laid a theoretical foundation for their translational application in skin health [19,20]. However, systematic sorting and summary on the formulation development and clinical efficacy validation of these bioactives remain insufficient, which limits their further practical application in skincare.

To address these identified gaps, the present review synthesizes findings from 129 studies on plant-derived bioactive compounds published between 2021 and 2025 across multiple databases, and establishes a complete research logical chain: plant-derived bioactive compounds → antioxidant/autophagy regulatory mechanisms → bifunctional synergistic effects in skin → translational research. Specifically, this review integrates classical and novel antioxidant targets, as well as general and selective autophagy targets, clarifies the functional features and regulatory mechanisms of bifunctional ingredients (with visual mechanistic support), and summarizes the progress of these compounds in cosmetic formulation development and clinical efficacy validation. The core value of this review lies in providing a targeted theoretical reference for mechanism-guided skin health research. It also supports the rational application and development of plant-derived bioactive compounds in molecular and translational skin studies.

## 2. Materials and Methods

Studies were identified via multiple academic databases, including PubMed, Web of Science, Scopus, and Wiley Online Library. To ensure the retrieval of relevant and targeted literature, specific keywords were used: “plant constituents”, “plant-based antioxidants”, “plant-derived bioactive compounds”, “plant extracts”, “botanical active compounds”, “botanical extracts”, “ROS scavenging”, “AMPK”, “MAPK”, “Nrf2”, “mTOR”, “autophagy”, “autophagosome formation”, “antioxidant defense”, “mitophagy”, “autophagy in skin protection”, “skin homeostasis”, “skin repair agents”, “skin”, and “translational applications”. These keywords were further combined using the Boolean operator “AND” as needed to enhance the comprehensiveness and accuracy of retrieval results. During the literature screening process, articles meeting the following criteria were excluded: those without full-text availability, redundant publications, personal opinions, studies irrelevant to the research focus (i.e., plant-derived bioactive compounds in skin protection and translational applications), letters to the editor, unpublished data, and non-English-language literature. The search was restricted to studies published between 2021 and 2025, ensuring this review reflects the latest research advances in the field. After initial retrieval and screening, a total of 129 core studies were ultimately included for comprehensive synthesis and analysis, based on their relevance to the review’s research framework, methodological rigor, and scientific impact.

## 3. Antioxidant Mechanisms of Plant-Derived Bioactive Compounds for Skin Protection

Skin exposure to external stressors easily induces excessive ROS accumulation and oxidative stress imbalance. Oxidative stress drives skin aging, barrier dysfunction, and pigment abnormalities [21,22,23]. Thus, effective antioxidant interventions for the skin are essential. Plant-derived bioactive compounds have become a primary source of natural antioxidants [5]. These compounds have structural diversity and functional versatility. They form a multi-level protective network via the principle of “structure determines activity, mechanism synergy, precise targeting”. This network includes three key antioxidant pathways: direct ROS scavenging, antioxidant enzyme activation, and core signaling pathway regulation [11]. It ultimately achieves skin lightening, anti-aging, and barrier repair effects. This provides a theoretical basis for the translational application of these compounds in skin care-related formulations.

### 3.1. Structure–Activity Relationship (SAR) of Plant-Derived Antioxidant Compounds

The antioxidant activity of plant-derived bioactive compounds ties closely to their chemical structures. Its core patterns focus on phenolic hydroxyl structural characteristics and modification regulation.

For compounds with the same parent nucleus, the phenolic hydroxyl number and position determine antioxidant strength. More hydroxyl groups usually enhance electron transfer and free radical scavenging. For example, gallic acid (algal polyphenols) has a stronger scavenging capacity than (−)-epicatechin [24]. This is due to gallic acid’s polyhydroxyl structure compared to (−)-epicatechin. Among flavonols, quercetin has higher activity than kaempferol. Quercetin has two hydroxyl groups at the B-ring 4′ and 5′ positions. Kaempferol only has one hydroxyl group at the B-ring 4′ position [25]. Notably, specific hydroxyl sites play more prominent roles in antioxidant activity. Anthocyanin C4′ hydroxyl is key for free radical scavenging [26]. Scutellarein 6-OH scavenges OOH^•^ via the hydrogen atom transfer (HAT) mechanism [27]. Herbacetin’s A-ring 7″/8′ hydroxyls inhibit cellular oxidative stress [28].

Structural modification has a dual regulatory effect on antioxidant activity. Esterification enhances catechin activity but reduces gallic acid’s ^•^OH scavenging [24]. Gallate esters (e.g., C_2_-GA, gallic acid ethyl ester) improve antioxidant efficiency via oil-phase hydrogen bonding [29]. Curcumin-pyrrole coupling enhances activity via polyphenol-electron-withdrawing synergy [30]. The ratio of structural units (syringyl (S), guaiacyl (G), and p-hydroxyphenyl (H)) in lignin can serve as a key indicator for screening high-efficiency antioxidants [31].

In summary, the core SAR of plant-derived antioxidant compounds can be summarized as “hydroxyl-dominated, site-enhanced, and modification-regulated”. These patterns guide the rapid screening and targeted optimization of natural antioxidants. Such guidance provides a molecular-level basis for the development and optimization of bioactive skin-protective agents.

### 3.2. Antioxidant Effects and Mechanisms of Plant-Derived Bioactive Compounds

Plant-derived bioactive compounds form a multi-pathway antioxidant network. These pathways include non-enzymatic intervention, enzymatic synergy, and signaling regulation. This network effectively addresses the skin’s complex oxidative stress environment.

#### 3.2.1. Non-Enzymatic Antioxidant Effect

The non-enzymatic effect is a “rapid response” for plant-derived compounds against oxidative stress. It does not rely on antioxidant enzymes for mediation. It acts directly on ROS or oxidative metabolic pathways to block damage.

This mechanism is mainly achieved through two key pathways.

Direct scavenging of ROS and oxidative intermediates: Phenolic acids (e.g., ascorbic acid, ferulic acid) neutralize particulate matter (PM) + UV-induced ROS. These ROS include superoxide anions and hydroxyl radicals. They also reduce lipid peroxidation products (e.g., 4-hydroxy-2-nonenal, 4HNE) [23]. This protects the integrity of skin barrier proteins. Pomegranate and sweet orange extracts scavenge H_2_O_2_-induced ROS efficiently. They act via HAT or electron transfer (ET) mechanisms. Their antioxidant efficiency correlates positively with total phenol content [32]. *Piper Betle* Linn. leaf methanol extract directly scavenges α,α-diphenyl-β-picrylhydrazyl (DPPH) free radicals. It also inhibits the nuclear factor kappa B (NF-κB) pathway to reduce inflammation-related ROS [33]. This gives it a dual “antioxidant + anti-inflammatory” effect.

Inhibiting ROS synthesis by interfering with oxidative metabolism: Black tea extract protects aconitase activity in the mitochondrial tricarboxylic acid (TCA) cycle. This maintains TCA cycle efficiency and ATP synthesis. It further reduces ROS from abnormal mitochondrial respiratory chains [34].

The non-enzymatic effect has a fast reaction rate and direct targeting. These traits enable rapid neutralization of acute ROS accumulation. They also lay the foundation for plant-derived compounds’ antioxidant defense.

#### 3.2.2. Enzymatic Synergistic Antioxidant Effect

The enzymatic synergistic effect is core to enhancing skin’s endogenous antioxidant capacity. It regulates key antioxidant enzymes (superoxide dismutase (SOD), catalase (CAT), and glutathione peroxidase (GSH-Px)) via activity or expression modulation. This strengthens the skin’s sustained resistance to oxidative stress.

In UVB damage models, unhydrolyzed collagen and epigallocatechin gallate EGCG (alone or combined) are pretreated. They restore UVB-reduced SOD and GSH-Px activities. They also lower lipid peroxidation product (malondialdehyde, MDA) levels. Notably, the combined effect outperforms single components [35]. *Dendrobium officinale* flower extract ameliorates D-galactose-induced skin aging. It achieves this by activating SOD and CAT activities [36]. Processed *Scutellaria baicalensis* extract (PSGE) upregulates heme oxygenase-1 (HO-1) and NAD(P)H: quinone dehydrogenase 1 (NQO_1_) expression. It simultaneously enhances anti-inflammatory and antioxidant effects [37].

In summary, plant-derived compounds compensate for non-enzymatic “short duration” limits. They activate or restore the antioxidant enzyme system. This enhances the stability and sustainability of endogenous antioxidant defense. It serves as a key mechanism for long-term skin antioxidant protection.

#### 3.2.3. Regulatory Mechanisms of Antioxidant Signaling Pathways

**Figure 2:**
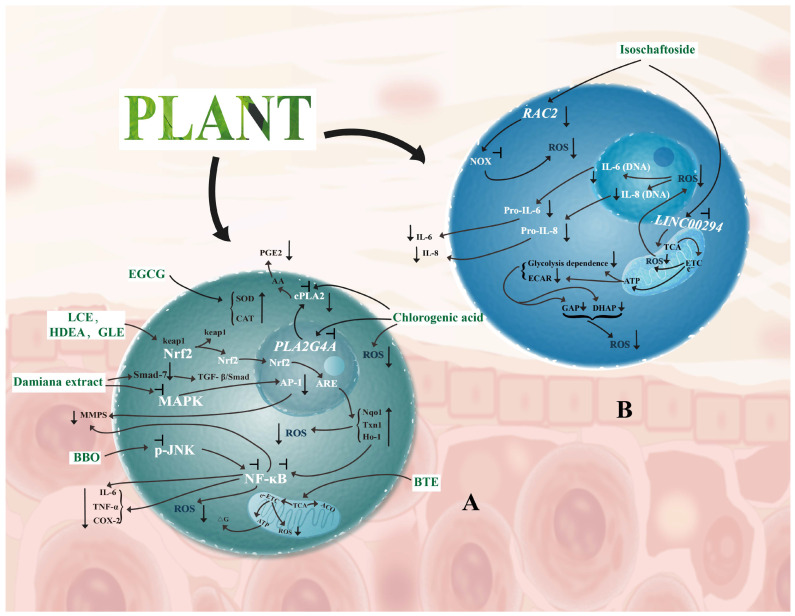
Signaling pathways associated with the antioxidant mechanisms of plant-derived bioactive compounds. (**A**) Classical antioxidant signaling pathways of plant bioactive compounds in cells; (**B**) non-classical antioxidant pathways of plant bioactive compounds. PLANT = plant-derived bioactive compounds; ↓ = downregulation or decrease; ↑ = upregulation or increase; **⊣** = inhibition; → = activation or action on the indicated target. Isoschaftoside; BTE: black tea PSR™ extract; LCE: *Lavandula angustifolia* leaf callus extract; HDEA: red alga *Halymenia durvillei* ethyl acetate fraction; GLE: ginkgo leaf extract; chlorogenic acid (from *Oenanthe javanica* extract); damiana extract]; BBO: *Blumea balsamifera* essential oil; EGCG: epigallocatechin gallate.

Antioxidant signaling pathways connect plant-derived compounds’ extrinsic and intrinsic defenses. They target key transcription factors or kinases to mediate cascade regulation. The cascade follows “signal activation–target gene expression–functional effect”. This is a classic mode of cellular antioxidant defense. Antioxidant pathways include classical and unconventional types (Figure 2).

##### Nrf2-ARE Pathway

The Nrf2-ARE pathway regulates the skin’s endogenous antioxidant defense centrally [38,39]. It follows a “resting inhibition–activation–translocation–target gene expression” pattern. Under quiescent conditions, Nrf2 binds to ECH-associated protein 1 (Keap1). Bound Nrf2 then undergoes proteasomal degradation. Oxidative stress or plant components trigger Nrf2 dissociation from Keap1. Nrf2 translocates to the nucleus and binds to antioxidant response elements (ARE) [40]. This initiates expression of SOD, CAT, HO-1 and other target genes. Ultimately, it enhances the skin’s antioxidant capacity.

Plant-derived compounds activate this pathway with distinct targeting. *Lavandula angustifolia* leaf callus extract (LCE) downregulates Keap1 expression. This promotes Nrf2 nuclear translocation and elevates glutathione (GSH) levels [41]. Harpagogenin (an acid-hydrolyzed product of *Stachys sieboldii* extract) activates ARE in a concentration-dependent manner. Its mechanism matches the positive control sulforaphane [42]. Sea buckthorn naringenin acts in UVB-induced skin photodamage models. It upregulates Nrf2 expression and catalase activity. It reduces mitochondrial fragmentation and ROS production. It also inhibits NF-κB phosphorylation and pro-inflammatory cytokine secretion [43]. Red alga *Halymenia durvillei* ethyl acetate fraction (HDEA) promotes Nrf2 translocation. It activates SOD2, heme oxygenase (HMOX) and other target genes synchronously. This reduces UV-induced ROS and matrix metalloproteinase 1/3 (MMP-1/3) expression. It further protects skin collagen structure [44].

Nrf2 pathway downstream effects translate directly to skin protection. Ginkgo leaf extract (GLE) activates Nrf2-ARE to upregulate HO-1 and GSH. This enhances keratinocyte antioxidant capacity. It also reduces collagen degradation and promotes normal desquamation. These actions exert anti-aging effects on the skin [45]. Modified Qing’e Formula (MQEF) activates the Nrf2 pathway. It reduces UVB-induced keratinocyte apoptosis. It inhibits abnormal MMP expression and epidermal thickening. It ameliorates elastic fiber fragmentation and maintains skin barrier function [46].

##### MAPK Pathway

The MAPK pathway regulates skin oxidative stress and photoaging pivotally [38]. UV-induced ROS bursts activate MAPK subfamilies (c-Jun N-terminal kinase (JNK), p38 mitogen-activated protein kinase (p38), extracellular signal-regulated kinase (ERK)). These activated kinases promote activator protein 1 (AP-1) and NF-κB nuclear translocation. They upregulate MMP expression and trigger inflammatory factor release [47]. These actions lead to collagen degradation and epidermal hyperplasia. Ultimately, they exacerbate skin photoaging. Plant-derived compounds block this cascade by inhibiting MAPK phosphorylation.

Multiple studies have validated this regulatory mechanism. Resveratrol inhibits UVB-induced p38 and ERK activation in HaCaT cells. It reduces MMP-1/9 expression and suppresses collagen degradation [48]. *Abeliophyllum distichum* leaf extract (AL) inhibits LPS-induced ERK1/2 phosphorylation. It acts on RAW264.7 cells without affecting MEK1/2 phosphorylation. AL downregulates NADPH oxidase 1/2 (NOX_1_/NOX_2_) and upregulates NQO_1_, γ-glutamyl cysteine synthetase (GCLC). It also suppresses inducible nitric oxide synthase (iNOS), interleukin-6 (IL-6), and cyclooxygenase 2 (COX-2) production [49]. Rosa davurica leaf extract (RDE) acts on UVB-exposed HaCaT cells. It inhibits ERK, JNK, and p38 phosphorylation. This reduces MMP-1/3 expression and mitigates collagen degradation [50].

MAPK inhibition by plant compounds translates to multidimensional skin protection. Macroscopically, it alleviates UV-induced dryness, erythema, and wrinkles. It also enhances skin hydration. Microscopically, it prevents collagen rupture and inflammatory stress. This maintains the skin’s structural integrity and functional homeostasis. The MAPK pathway complements the Nrf2 pathway functionally. Together, they form the core of the classical antioxidant network.

##### Novel Targets and Unconventional Regulatory Mechanisms

Traditional antioxidant research mainly focuses on Nrf2 and MAPK pathways. Glycosidic flavonoids (e.g., isoschaftoside) expand antioxidant functional dimensions. They act via unconventional pathways: “novel molecular targeting + metabolic reprogramming” [13].

Targeted regulation of cross-boundary novel molecules: Rac family small GTPase 2 (*RAC2*) is traditionally linked to phagocytic oxidative bursts. Recent studies identify it as an activator of NOX in senescent cells. Downregulating *RAC2* inhibits ROS synthesis at the source. Long intergenic non-protein-coding RNA 294 (*LINC00294*) was once studied in oncology. New evidence shows it amplifies oxidative stress via epigenetics in senescent cells [13]. Inhibiting *LINC00294* alleviates inflammation-related oxidative stress. It also regulates mitochondrial function. This establishes an “RNA–mitochondria” cross-boundary regulatory link [13]. Isoschaftoside precisely targets both *RAC2* and *LINC00294*. It demonstrates the multi-dimensional intervention advantages of plant-derived compounds.

Metabolic reprogramming-mediated novel antioxidant pathway: The classical Nrf2 pathway focuses on downstream ROS scavenging. Isoschaftoside adopts a “source control–metabolic repair” strategy [13]. On one hand, it inhibits *RAC2* to reduce ROS production. On the other hand, it repairs mitochondria and enhances respiratory chain efficiency. This decreases mitochondria-derived ROS. It also reduces cellular dependence on glycolysis. This alleviates oxidative stress at the metabolic root.

Structure–activity advantages of glycosidic flavonoids: Glycosidic flavonoids (e.g., isoschaftoside, astragalin) have superior ROS scavenging efficacy. Glycosyl substitution enhances their molecular stability and cellular permeability [13]. This breaks the paradigm that flavonoids “rely solely on hydroxyls for activity”. It provides a new molecular basis for antioxidant component screening.

Relevant research on this innovative model is still in its infancy. It offers key insights for the precise development of plant-derived antioxidants. It also expands the research boundaries of antioxidant mechanisms.

##### Multi-Pathway Crosstalk, Synergy, and Network Regulation

Plant-derived bioactive compounds exert high-efficiency antioxidant activity via multi-pathway crosstalk. They form a “multi-target synergy, full-chain protection” network regulation mode. This is their key advantage in addressing the skin’s complex oxidative stress.

“Antioxidant–anti-inflammatory” bidirectional inhibition: Oxidative stress and inflammation often exacerbate each other mutually. Plant-derived compounds target cross-regulatory nodes for bidirectional inhibition. Schisandra chinensis extract scavenges PM_2.5_-induced ROS. It blocks NF-κB activation and reduces iNOS/COX-2 expression [51]. Chlorogenic acid (from *Oenanthe javanica* extract) acts on PM_10_-damaged skin cells. It directly scavenges ROS to reduce lipid peroxidation. It inhibits phospholipase A2 group IVA (*PLA2G4A*) transcription and cytosolic phospholipase A2 (cPLA2) phosphorylation. This blocks arachidonic acid release upstream. It further reduces pro-inflammatory prostaglandin E2 (PGE2) synthesis [52]. PSGE activates the Nrf2/HO-1 pathway. HO-1 inhibits the inhibitor of nuclear factor kappa B alpha (IκBα) phosphorylation and NF-κB nuclear translocation. This forms a “antioxidation→anti-inflammation→reduced ROS” virtuous cycle [37].

“Stress defense–collagen maintenance” functional complementarity: Skin oxidative stress is often accompanied by collagen degradation. Plant-derived compounds maintain collagen homeostasis via “inhibition + repair” synergy. Damiana extract inhibits p38/ERK/JNK phosphorylation. This reduces AP-1-mediated MMP-1/3 expression (collagen degradation inhibition). It also downregulates small mothers against decapentaplegic homolog 7 (Smad-7) to activate the transforming growth factor-beta (TGF-β)/Smad pathway. This promotes type I procollagen synthesis (collagen production enhancement) [53]. *Emblica officinalis*–barley sprout complex inhibits JNK/c-Fos/c-Jun pathway. It reduces MMP expression and alleviates UVB-induced wrinkles. It simultaneously activates the TGF-βR1/Smad3 pathway to upregulate collagen synthesis [54]. *Lithospermum-Lobata* complex (LP) acts on UVB-exposed HaCaT cells. It inhibits the MAPK/NF-κB pathway to reduce collagen degradation. It also activates the TGF-β pathway to promote type I procollagen synthesis [55].

“Core pathway hub-multi-effect hierarchical regulation”: Plant-derived compounds act as upstream hubs in key pathways for multi-effect synergy. *Blumea balsamifera* essential oil (BBO) acts on human dermal fibroblasts (HDF). It inhibits JNK/MAPK phosphorylation and NF-κB nuclear translocation. It reduces inflammatory factors, MMPs, and senescence marker activity. In vivo, it enhances antioxidant enzyme activity and mitigates UVA damage [56]. Black tea PSR™ extract preserves mitochondrial aconitase activity. It maintains metabolic homeostasis and scavenges ROS. It also repairs DNA and protects the extracellular matrix (ECM). Its ECM regulatory effect is superior to coenzyme Q10 (CoQ10) [34].

In summary, functional integration is core to multi-pathway network regulation. It breaks vicious cycles and targets core needs via multi-effect hierarchical regulation, forming a “prevention–clearance–repair” comprehensive protective network. Such multi-target regulatory characteristics may render plant-derived compounds a complementary option to single-target chemical drugs in addressing complex skin oxidative stress-related issues.

### 3.3. Skin Effects of Plant-Derived Bioactive Compounds via Antioxidation

Plant-derived bioactive compounds regulate skin oxidative stress balance. They exert multidimensional skin protection effects (lightening, anti-aging, barrier repair). “Antioxidation” serves as the core mediating pathway here. This reflects high consistency between “mechanism and effect”.

#### 3.3.1. Skin Lightening and Pigment Homeostasis Regulation

Oxidative stress is a key inducer of abnormal melanin synthesis. Plant-derived compounds inhibit excessive melanin via antioxidation. They regulate skin pigment homeostasis and fade melasma [57]. Agarooligosaccharides (AO) are from the red alga Gracilaria lemaneiformis. They scavenge ROS in B16F10 melanoma cells. This reduces oxidative stress-induced melanocyte activation. AO also directly inhibits tyrosinase activity. It blocks alpha-melanocyte-stimulating hormone (α-MSH)-induced melanin synthesis [58]. *Emblica officinalis*–barley malt complex (IB) scavenges UVB/3-isobutyl-1-methylxanthine (IBMX)-induced ROS. It reduces cyclic adenosine monophosphate (cAMP) levels to inhibit protein kinase A (PKA)/cAMP-responsive element-binding protein (CREB) pathway activation. This downregulates melanocyte-inducing transcription factor (MITF), tyrosinase, and tyrosinase-related protein 1/2 (TRP-1/2) expression. Ultimately, it decreases melanin production [54]. Tanshinone I (T-I) and dihydrotanshinone (DHT) are tanshinones. They activate the Nrf2 pathway in human epidermal melanocytes (HEM). This reduces UVA-induced ROS levels. It further downregulates MITF and tyrosinase expression. Nrf2 knockdown eliminates the skin lightening effect. This confirms that antioxidation is core to melanin inhibition [59].

#### 3.3.2. Anti-Aging and Photoprotection

UV (UVA/UVB)-induced ROS accumulation drives skin aging primarily. Plant-derived compounds delay skin aging via two dimensions. These are structural maintenance and functional repair. They exert effects via the cascade: “ROS scavenging–collagen protection–cellular senescence inhibition”.

Collagen protection: Plant-derived compounds inhibit collagen degradation and promote synthesis. *Garcinia mangostana* peel extract (MPE) acts on primary human dermal fibroblasts (PHDF). It reduces UVA-induced ROS accumulation. It downregulates MMP-1 and increases type I procollagen synthesis. This alleviates wrinkles and reduces skin elasticity [60]. *Artemisia frigida* essential oil (AEO) has both UVB filtering and antioxidant activities. It inhibits MMP-1/3 expression and reduces collagen degradation. It also decreases hydroxyproline loss. This improves epidermal thickening and abnormal elastic fibers [61].

Cellular senescence inhibition: Plant-derived compounds protect cells from oxidative damage. EGCG acts on UVA-induced human skin fibroblasts (HSF). It reduces senescence-associated β-galactosidase (SA-β-gal)-positive cells. It protects telomeres from oxidative damage. EGCG upregulates SOD and CAT activities. It promotes transforming growth factor-beta1 (TGF-β1) secretion and inhibits MMP expression. This maintains extracellular matrix (ECM) homeostasis [62]. *Phyllanthus emblica* extract (PE) counteracts UVA damage via the ERK/TGF-β/Smad pathway. It upregulates collagen and restores antioxidant enzyme activity. PE inhibits the MAPK/AP-1 pathway in UVB-damaged cells. It reduces inflammatory factors and apoptosis markers. This achieves both anti-aging and damage repair [63].

#### 3.3.3. Skin Barrier Protection and Repair

The skin barrier (stratum corneum, intercellular lipids) is vulnerable to ROS-induced inflammation. Plant-derived compounds restore barrier homeostasis via synergistic effects. These effects follow “antioxidation–anti-inflammation–barrier repair”.

Structural maintenance: Plant-derived compounds protect key barrier components via antioxidation. Ascorbic acid–ferulic acid–tocopherol complex (CF Mix) scavenges PM + UV-induced ROS. It prevents downregulation of loricrin and filaggrin (key barrier proteins). It also maintains normal keratinocyte differentiation. This avoids stratum corneum structural disorders [23]. Rosmarinic acid (RA) upregulates sodium proton exchanger 1 (NHE1). This restores the skin’s physiological acidic environment. RA promotes ceramide synthesis and reduces transepidermal water loss (TEWL) [64].

Damage repair: Plant-derived compounds alleviate damage and promote barrier regeneration. This is achieved through antioxidation. *Lavandula angustifolia* leaf callus extract (LCE) activates the Nrf2 pathway. It enhances skin barrier integrity and reduces PM adhesion. It also diminishes skin redness. This exerts “antioxidation–barrier strengthening–pollutant defense” effects [41]. *Chlorella pyrenoidosa* extract (CPE) chelates PM_2.5_ heavy metal ions. It reduces oxidative damage to skin cells. CPE repairs DNA breaks and restores cell proliferation. This maintains skin barrier homeostasis [65].

In summary, the antioxidant effect of plant-derived bioactive compounds conforms to a “structure–mechanism–effect” logical chain. Their advantages include natural origin, multi-functionality, and network regulation for complex skin oxidative stress. This addresses some of the limitations of single-target chemical antioxidants and provides insights into the rational design of natural skin-protective agents.

## 4. Autophagy-Regulating Mechanisms of Plant-Derived Bioactive Compounds for Skin Protection

Plant-derived bioactive compounds are important natural ingredients for skin protection. Antioxidation is one of their well-established core mechanisms. Skin protection also requires clearance of accumulated cellular damage. Such damage cannot be fully addressed by antioxidant mechanisms alone. Autophagy serves as an independent and essential skin protective pathway [15,66]. It is a “selective degradation and recycling” process in skin cells. It specifically removes dysfunctional organelles, misfolded proteins and lipofuscin. It also regulates epidermal renewal, collagen homeostasis and skin barrier function. This is crucial for maintaining skin health and resisting external insults [67,68].

However, autophagic function is vulnerable to disruption. Extrinsic stimuli such as UV irradiation and environmental pollution can induce autophagic imbalance. Imbalance may present as insufficient autophagic activity. This leads to incomplete clearance of damaged substances and accelerated skin aging. It may also manifest as excessive autophagic activation. This causes unnecessary cellular injury and impaired skin function [69]. Both forms of imbalance contribute to skin issues such as hyperpigmentation and roughness.

Plant-derived bioactive compounds possess natural advantages for regulating autophagy. They are multi-targeted and low in toxicity. These traits make them ideal for modulating autophagic function. They act on key autophagic signaling pathways in skin cells. Such pathways include adenosine 5′-monophosphate-activated protein kinase (AMPK)/mammalian target of rapamycin (mTOR) and MAPK [7,14,66]. These pathways are critical for autophagic initiation and progression.

For underactive autophagy, plant-derived bioactive compounds promote autophagic activation. This enhances clearance of accumulated cellular damage. For overactivated autophagy, they inhibit excessive autophagic responses. This protects skin cells from unnecessary injury. Through precise regulation, these compounds restore balanced autophagic function in skin cells [67,68,70].

Restored autophagy further translates into specific skin protective effects. These effects include alleviating hyperpigmentation, improving skin smoothness and delaying photoaging. They also enhance skin barrier repair and collagen synthesis [15,66,68]. Plant-derived bioactive compounds’ autophagy-regulating capacity enhances their translational potential for skin health and protection.

### 4.1. Core Mechanisms of Plant-Derived Bioactive Compounds in Regulating Skin Cell Autophagy

**Figure 3:**
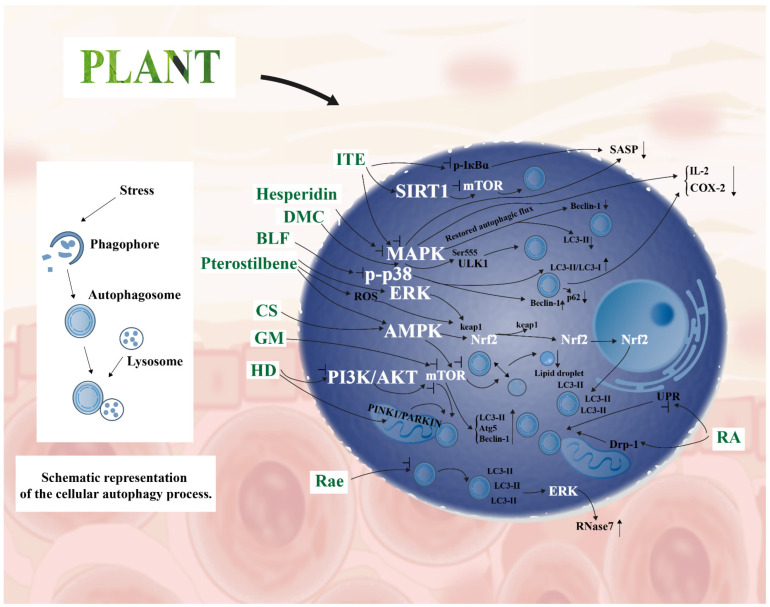
Signaling pathways associated with the autophagy mechanisms of plant-derived bioactive compounds. PLANT = plant-derived bioactive compounds; ↓ = downregulation or decrease; ↑ = upregulation or increase; **⊣** = inhibition; → = activation or action on the indicated target. RA: rosmarinic acid; pterostilbene; GM: γ-Mangostin; HD: *Hedyotis diffusa* extract; DMC: 4,4′-Dimethoxychalcone; BLF: bamboo leaf flavonoids. Hesperidin; ITE: *Isatis tinctoria* leaf extract; CS: Camellia saponins; Rae: *Ruscus aculeatus* extract.

Plant-derived bioactive compounds regulate cellular autophagy by modulating core signaling pathways and selective autophagy. This contributes to cellular homeostasis and protects against skin damage induced by autophagic imbalance. The relevant signaling pathways are illustrated in Figure 3.

#### 4.1.1. AMPK/mTOR Pathway

AMPK (energy sensor) and mTOR (nutrient sensor) interaction maintains autophagic homeostasis. Plant compounds restore autophagic imbalance via two approaches. These are “activating AMPK” or “inhibiting mTOR” for abnormal energy metabolism.

AMPK-mediated autophagy activation: Rose extract (RE) induces AMPK phosphorylation. It upregulates autophagy-related molecules and delays cellular senescence. An AMPK-specific inhibitor completely blocks this effect. This confirms AMPK’s key role in energy-dependent autophagy [71]. Pterostilbene acts on HaCaT cells. It activates ERK, AMPK pathways, and ROS signaling. It induces Nrf2 nuclear translocation. It promotes microtubule-associated protein 1 light chain 3-II (LC3-II) accumulation and autophagic vacuole (AVO) formation. This initiates the autophagic process [72].

mTOR inhibition: γ-Mangostin (GM) relieves UVB-induced excessive mTOR activation. It upregulates LC3-II, autophagy-related protein 5 (Atg5), and Beclin 1 expression. It accelerates autophagosome formation and reduces collagen degradation. This maintains skin matrix homeostasis [73]. *Hedyotis diffusa* extract (HD) targets two pathways. These are PI3K/AKT/mTOR and PTEN-induced kinase 1 (PINK1)/RBR E3 ubiquitin protein ligase (PARKIN) pathways. It alleviates UVB-induced HaCaT cell autophagy imbalance. It also inhibits UVB-induced HaCaT cell apoptosis [74].

In summary, the core rule is “energy/nutrient-guided autophagic repair”. Plant compounds regulate AMPK/mTOR to match stress-induced energy needs. This restores autophagic function, clearing damage and re-establishing homeostasis.

#### 4.1.2. MAPK Pathway

The MAPK pathway integrates external stress signals (oxidative stress, inflammation). It acts as a core hub for skin cells. Plant-derived compounds restore “autophagy–inflammation” crosstalk imbalance via this pathway. This avoids stress-induced skin damage.

Repair of insufficient autophagy via activation: 4,4′-Dimethoxychalcone (DMC) is derived from *Angelica sinensis*. It activates the MAPK pathway in 2,2′-azobis(2-amidinopropane) dihydrochloride (AAPH)-induced senescent HaCaT cells. This promotes phosphorylation of unc-51-like autophagy activating kinase 1 (ULK1, Ser555). ULK1 is a key protein for autophagy initiation. DMC induces autophagosome assembly and clears senescence-related damage [75]. Bamboo leaf flavonoids (BLF) mainly contain orientin and isoorientin. They act on the HaCaT cell senescence model. They target the p38 MAPK subfamily and inhibit its phosphorylation. This upregulates the LC3-II/LC3-I ratio and Beclin-1 expression. It also downregulates sequestosome 1 (p62/SQSTM1). BLF activates autophagy and reduces IL-2 and COX-2 levels. This achieves “autophagy activation–anti-inflammation” synergistic repair [76].

Regulation of abnormal autophagy: PM_2.5_ induces excessive MAPK (p-ERK/p-JNK/p-p38) activation. This leads to “non-protective autophagy + inflammatory burst”. Hesperidin inhibits this excessive MAPK activation. It downregulates abnormal Beclin-1 and LC3-II levels in HaCaT cells. It reduces pro-inflammatory factors and apoptotic proteins. This restores HaCaT cell viability [77]. *Isatis tinctoria* leaf extract (ITE) acts on human dermal fibroblasts (RS-HDFs). It reduces p-JNK, p-ERK, and phosphorylated inhibitor of nuclear factor kappa-B alpha (p-IκBα) levels. It blocks chronic inflammation-associated senescence-associated secretory phenotype (SASP) release. It also restores autophagy via the sirtuin 1 (SIRT1)/mTOR pathway. This clears senescent damage in RS-HDFs [78].

In summary, the core mechanism is “stress signal integration + balanced repair”. Plant compounds regulate MAPK to repair insufficient autophagy. They also inhibit excessive autophagy and alleviate inflammation. This forms an “autophagy regulation–anti-inflammation” virtuous cycle.

#### 4.1.3. Selective Autophagy

Selective autophagy is a targeted degradation process that identifies and removes damaged organelles (e.g., mitochondria), abnormal lipids, and other subcellular components. It thereby enables precise cellular repair and preserves cellular homeostasis in skin cells.

Mitophagy repair: *Hedyotis diffusa* extract (HD) acts on UV-induced senescent HaCaT cells. It activates PINK1/PARK2-mediated mitochondrial labeling. This promotes autophagosome encapsulation of damaged mitochondria. HD also inhibits phosphatidylinositol 3-kinase (PI3K)/protein kinase B (AKT)-mediated mTOR activation. This relieves autophagic suppression [74]. Rosmarinic acid (RA) acts on primary human dermal fibroblasts (HDFs). It alleviates UVB-induced endoplasmic reticulum stress. It reverses abnormal dynamin-related protein 1 (Drp-1) expression. This restores mitophagy function [17]. Polydatin (PLD) acts on Down syndrome human skin fibroblasts (DS-HSFs). It downregulates chromosome 21-encoded miR-155. This relieves miR-155’s inhibition on mitochondrial transcription factor A (TFAM). TFAM is a mitochondrial transcription factor A. PLD enhances mitophagy and improves mitochondrial dysfunction. This expands plant compounds’ mechanistic diversity via non-coding RNAs [18].

Lipophagy: Camellia saponins activate the AMPK/mTOR pathway. They act on human sebocytes (SZ95). They promote autophagosome–lysosome fusion. This restores oleic acid/linoleic acid (OL)-induced autophagic flux blockage. It accelerates lipid droplet degradation. This alleviates OL-stimulated sebocyte lipid accumulation [79].

In summary, the core feature is “targeted subcellular damage clearance”. Plant compounds modulate selective autophagy to eliminate damaged organelles/lipids. This preserves intact cellular structures and maintains subcellular homeostasis.

#### 4.1.4. Bidirectional Regulation of Autophagy

Autophagy’s impact on skin health depends on “dynamic adaptation” [7]. A core advantage of plant-derived bioactive compounds lies in bidirectional autophagy regulation. This regulation is based on skin physiological needs or pathological states [68,70]. They selectively inhibit autophagy in specific scenarios. They also activate underactive autophagy when necessary. This avoids cellular damage from excessive autophagic activation. It also prevents damage accumulation due to insufficient autophagy. This reflects natural compounds’ specificity and flexibility in autophagy regulation.

Autophagy inhibition: *Ruscus aculeatus* extract (RAE) selectively inhibits late-stage autophagy. It induces LC3-II accumulation and autophagosome retention. This supports ERK activation and ribonuclease 7 (RNase 7) expression. RNase 7 is an antimicrobial peptide. This enhances skin defense-related antimicrobial peptide expression. It further supports skin health protection [80]. Oxymatrine inhibits autophagic activity in HSFs. It reduces cell viability and collagen metabolism. It also induces HSF apoptosis. In vivo models show that it reduces hypertrophic scar area. It achieves this via autophagy inhibition. It downregulates Col I and Collagen Type I (Col I) and alpha-smooth muscle actin (α-SMA) (scar markers). This provides an autophagy-regulating strategy for scar repair [81]. A compound from the rhizome of *Acorus calamus var. angustatus* *Besser* inhibits Beclin-1. Its structure is (−)-syringaresinol 4-O-β-D-apiofuranosyl-(1→2)-β-D-glucopyranoside. It inhibits Beclin-1-mediated autophagic processes. It reduces pro-inflammatory factor release in LPS-induced cells [82].

Repair of damaged autophagy: Rosmarinic acid (RA) acts on primary HDFs. It alleviates UVB-induced endoplasmic reticulum stress. It restores mitophagy function. It reduces intracellular damage and mouse skin photodamage [17]. Shikimic acid (SA) acts via an SIRT1-dependent pathway. It upregulates chaperone protein binding immunoglobulin protein (BiP) expression. It inhibits abnormal unfolded protein response (UPR) activation. This reduces UPR-dependent excessive autophagy. It repairs UV-induced autophagic imbalance. It also avoids misfolded protein accumulation [83].

In summary, the core mechanism is “skin scenario-specific adaptation”. Plant compounds target specific autophagic nodes and act in a need-based manner: antimicrobial defense, scar repair, inflammation alleviation, UV damage repair, and autophagic imbalance correction. This achieves “adaptive inhibition” or “activating repair”, which aligns with skin physiological homeostasis and addresses skin functional impairments in a targeted manner. This underscores the distinctive advantages of natural compounds in modulating autophagy for skin care applications.

### 4.2. Skin Effects of Plant-Derived Bioactive Compounds Mediated by Autophagy

Plant-derived compounds regulate autophagy to exert distinct skin protective effects. They focus on core skin needs (pigment homeostasis, senescent clearance, barrier repair). The core logic is “repair autophagic imbalance, re-establish homeostasis”. This reflects consistency between “mechanism and effect”.

#### 4.2.1. Skin Lightening and Pigment Regulation

Plant-derived compounds restore melanin metabolism-related autophagic imbalance. The two main modes are autophagy-mediated melanosome degradation and the autophagy-signaling pathway synergistic melanin synthesis inhibition. These modes maintain pigment homeostasis and prevent hyperpigmentation or hypopigmentation.

Melanosome degradation: *Camellia sinensis* (tea leaf)-derived nanovesicles (TLNVs) regulate autophagy. They upregulate LC3B expression and downregulate p62 levels. TLNVs contain miR-828b that targets Myeloblastosis transcription factor 4 (MYB4). This inhibits the MITF/tyrosinase pathway. TLNVs penetrate the dermis up to 200 μm. They outperform traditional tea extracts in UVB-induced hyperpigmentation mice [84]. Resveratrol activates the Nrf2–Transcription Factor EB (TFEB)/TFE3 axis. It enhances the autophagy–lysosomal system activity. This promotes melanosome clearance in human dermal fibroblasts (HDFs). It repairs hyperpigmentation from insufficient melanosome degradation [85].

Synergy of melanin synthesis inhibition and degradation: Apigenin–phloretin combination suppresses the Wingless/Integrated (Wnt) pathway. This reduces melanin production. It also activates autophagy (upregulates LC3, downregulates p62). This accelerates melanosome degradation [86]. Apigenin alone activates the PINK1/PARKIN pathway. It improves mitochondrial function and reduces mitochondrial ROS. This maintains melanocyte metabolic homeostasis. It also restores autophagy–mitochondrial functional imbalance [87].

#### 4.2.2. Anti-Aging and Photoprotection

Plant-derived compounds achieve anti-aging and photoprotection via two core mechanisms. One mechanism is activating autophagy to clear senescent damage. Such damage includes lipofuscin and damaged mitochondria. The other is repairing collagen metabolic disorders from autophagic imbalance. This dual regulation meets advanced anti-aging demands. These demands involve “clearing existing damage + delaying new damage”.

Senescent damage clearance: *Myrothamnus flabellifolia*-*Coffea arabica* seed extract (MflCas) acts on HDFs. It downregulates mTOR and upregulates Park2 and LC3B. This enhances HDF autophagic activity. It reduces lipofuscin accumulation and protein oxidative damage. This repairs aging from impaired autophagy [88]. Handelin is an active component of *Chrysanthemum indicum* extract. It activates autophagy in UVA-exposed HDFs. This reduces UVA-induced ROS generation. It decreases SA-β-gal-positive cells and Cyclin-dependent kinase inhibitor 1A (p21) expression. This protects HDFs from photoaging [89]. Isoschaftoside upregulates LC3B activity. It increases autophagosome formation. It clears lipofuscin and dysfunctional mitochondria. This breaks the “oxidative stress-accelerated aging” cycle [13]. Shikimic acid (SA) repairs UV-induced excessive autophagy. It reduces misfolded protein accumulation. It also inhibits cellular senescence [83].

Collagen metabolism regulation: Syringaresinol (SYR) is derived from *Panax ginseng* berries. It acts on H_2_O_2_-induced HaCaT cells. It upregulates LC3B and inhibits MMP-2/9 expression. This reduces collagen degradation [90]. *Corydalis heterocarpa* extract (CHE) acts on HDFs. It promotes autophagic flux (LC3B puncta, enhanced lysosomal activity). It downregulates senescence markers. This maintains collagen homeostasis [91].

#### 4.2.3. Skin Protection and Barrier Repair

Plant-derived compounds achieve barrier protection and repair via two core mechanisms: repairing autophagic imbalance to resist external stressors, and maintaining barrier structural integrity and homeostasis. These properties render plant-derived compounds well-suited for skin barrier–vulnerable conditions, including sensitive skin and pollution-induced skin damage.

Damage resistance: Lycium barbarum polysaccharide (LBP) acts on PM_2.5_-exposed HaCaT cells. It inhibits PM_2.5_-induced excessive autophagy. This reduces apoptosis from oxidative and endoplasmic reticulum stress. It further protects skin cells [92]. *Codonopsis pilosula* extract (CPE) activates autophagy. It reduces H_2_O_2_-induced melanocyte apoptosis. It maintains MITF expression and cell differentiation function. This avoids oxidative stress-induced barrier cell dysfunction [93].

Structural homeostasis maintenance: Apigenin synergistically maintains structural homeostasis at the organelle, cellular, and microenvironmental levels. In B16F10 melanocytes, apigenin activates PINK1/PARKIN-mediated mitophagy and corrects mitochondrial fusion–fission imbalance. It also upregulates Epithelial Cadherin (E-Cadherin) and alleviates dendritic atrophy [87]. In human primary melanocytes, apigenin induces the expression of autophagy-related genes. This maintains the structural homeostasis of skin-related cells [86]. In fibroblasts, apigenin can induce Dickkopf 1 (DKK1) expression and regulate extracellular matrix-related genes. This improves microenvironmental stability [86].

In summary, plant-derived compounds exert diverse skin-protective effects (pigment regulation, anti-aging, barrier repair) via autophagy modulation. Their core advantages lie in targeted regulation and bidirectional repair capacity, which enable the restoration of autophagic homeostasis in different skin physiological and stress scenarios. This provides a mechanistic foundation for developing plant-based skin care interventions targeting autophagy.

## 5. Dual Regulation of Antioxidant and Autophagic Pathways by Plant-Derived Bioactive Compounds for Skin Protection

Certain plant-derived bioactive compounds exert dual regulatory effects on antioxidant and autophagic pathways. These compounds can maintain skin homeostasis by coordinating damage intervention and damage clearance [94,95]. They possess multi-target regulatory properties and act as key mediators between antioxidant and autophagic signaling. On the one hand, they suppress oxidative stress by inhibiting ROS production and scavenging accumulated ROS, thus limiting the occurrence and spread of skin damage. On the other hand, they trigger autophagy to degrade ROS-damaged intracellular components, thereby restoring cellular homeostasis [13,96]. Through dual regulation of these two pathways, such plant-derived components establish a complementary and synergistic protective network. This coordinated mode of action effectively strengthens skin defense and supports the development of plant-based skincare agents targeting antioxidant–autophagy crosstalk.

### 5.1. Plant-Derived Bioactive Compounds with Dual Antioxidant and Autophagy-Regulating Properties

Dual-function plant-derived compounds are categorized by their structural characteristics and component composition into three groups. These groups exert dual antioxidant and autophagy-regulating effects via three distinct modes: “single-molecule independent bearing”, “multi-component co-bearing”, and “specific-type molecule targeted bearing”. Collectively, these compounds form a dual-functional component library that spans a wide range of skincare application scenarios.

#### 5.1.1. Flavonoid and Polyphenolic Monomeric Compounds

Flavonoid and polyphenolic monomers feature “single-molecule dual activities”. They possess functional groups such as phenolic hydroxyls and glycosidic bonds. These groups enable antioxidant and autophagic regulatory functions. The antioxidant function involves scavenging ROS or inhibiting its generation. The autophagic function focuses on activating targeted pathways/molecules. The two mechanisms are independent yet synergistic. This enhances overall skin protection efficacy.

Isoschaftoside (glycosidic flavonoid) acts on skin cells. It downregulates *RAC2* to inhibit NADPH oxidase activity. It inhibits *LINC00294* to improve mitochondrial function. This reduces upstream ROS generation. It also upregulates LC3B activity and promotes mitophagy. This clears lipofuscin and dysfunctional mitochondria. A “reducing ROS source + clearing damaged mitochondria” complement is formed [13].

Ginsenoside Rg2 (steroidal saponin) inhibits D-galactose-induced ROS. It reduces MDA levels in relevant models. It activates the AMPK pathway and upregulates the LC3-II/LC3-I ratio. It also increases Beclin-1 expression to enhance autophagic flux. This supports skin fibroblast anti-aging. It maintains porcine mesenchymal stem cell viability [97].

Curcumin (polyphenol) acts on UVB-exposed HaCaT cells. It directly scavenges UVB-induced ROS to alleviate acute damage. It targets Yes-associated protein 1 (YAP1) to activate mitophagy. This degrades damaged mitochondria and reduces cell apoptosis [96].

Syringaresinol (lignin) scavenges DPPH and 2,2′-azino-bis-(3-ethylbenzothiazoline-6-sulfonic acid) (ABTS) free radicals. It reduces H_2_O_2_-induced oxidative stress in HaCaT cells. It upregulates LC3B protein expression to activate autophagy. These effects synergistically inhibit H_2_O_2_-induced MMP-2/9 expression. This combats oxidative stress-related skin laxity and wrinkles [90].

Polydatin (stilbenoid polyphenol) acts on DS-HSFs. It reduces oxidative damage in these cells. It regulates the miR-155/TFAM pathway to activate mitophagy. This repairs dysfunctional mitochondria. It synergistically inhibits premature cellular senescence [18].

#### 5.1.2. Plant Extracts

Plant extracts feature “multi-component co-bearing dual activities”. They exert dual antioxidation–autophagy effects in two ways. One way is the direct action of specific active substances in extracts. Some components scavenge ROS, while others activate autophagy directly. The other way is autophagic clearance of ROS generation sources. These sources include damaged mitochondria and excessive lipid droplets. This auxiliarily alleviates oxidative stress.

*Corydalis heterocarpa* extract (CHE) contains rutin and iridin. These are key antioxidant components. CHE upregulates leucine-rich repeat and sterile alpha motif-containing 1 (LRSAM1) to activate autophagic flux. It clears damaged mitochondria, reducing ROS generation. It also downregulates p53 and p21 (senescence markers). This achieves “antioxidation + autophagic senescent clearance” anti-aging [91].

*Hedyotis diffusa* extract (HD) acts on UVB-exposed HaCaT cells. It reduces UVB-induced ROS accumulation (antioxidation). It inhibits the PI3K/AKT/mTOR pathway. It also activates PINK1/PARK2-mediated mitophagy. This maintains mitochondrial membrane potential. It synergistically alleviates skin barrier damage [74].

*Nelumbo nucifera* sprout extract (LSE) is rich in neferine and liensinine. These bisbenzylisoquinoline alkaloids have antioxidant activity [98,99]. LSE acts on MNT-1 human melanoma cells. It increases autophagic flux (LC3-II accumulation, autophagosome formation). This promotes melanosome degradation. It shows potential as a natural skin lightening agent [100].

Oleaster fruit extract is rich in oleuropein aglycone (F6 fraction). It has strong DPPH and ABTS free radical scavenging capacity. It induces autophagic activity in human foreskin fibroblasts (HFF). This protects cells from oxidative damage and enhances damage clearance. It is suitable for anti-aging cosmeceutical applications [101].

#### 5.1.3. Other Functional Compounds

This category features “specific-type molecule bearing dual activities”. They are mostly single-type natural molecules (polysaccharides, saponins). Their dual functions focus on reducing oxidative stress. They also regulate autophagic homeostasis for specific skincare needs.

Lycium barbarum polysaccharide (LBP) acts on PM_2.5_-exposed HaCaT cells. It reduces MDA content and increases SOD activity. It inhibits endoplasmic reticulum stress and regulates autophagic flux. This reduces cell apoptosis and maintains skin barrier homeostasis [92].

Camellia saponins (CS) activate AMPK and inhibit mTOR. This restores OL-induced autophagic flux blockage. They maintain a lysosomal acidic environment. They also promote autophagosome–lysosome fusion. This accelerates lipid droplet degradation. It indirectly lowers lipid peroxidation (oxidative stress source). This achieves “oil control + antioxidation” synergy [79].

### 5.2. Crosstalk-Mediated Synergistic Regulation of Antioxidant and Autophagy Pathways

**Figure 4:**
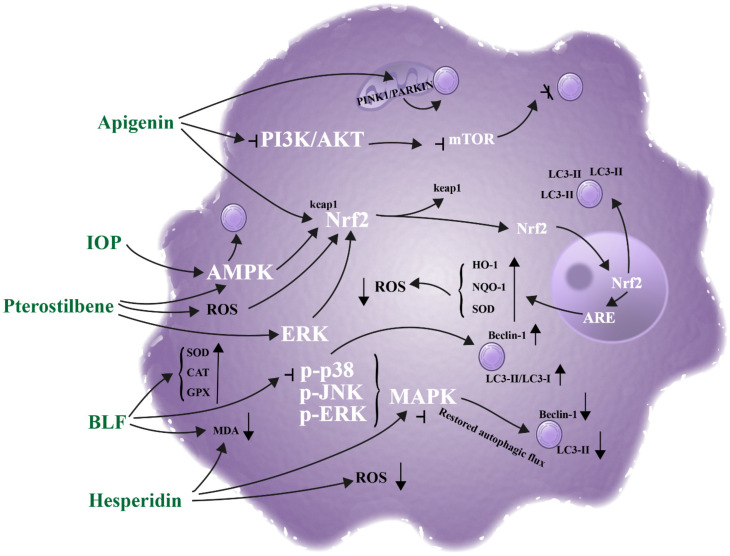
Signaling pathway crosstalk between antioxidation and autophagy regulated by plant-derived bioactive compounds. ↓ = downregulation or decrease; ↑ = upregulation or increase; **⊣** = inhibition; → = activation or action on the indicated target; **⊣** (with a slash above) = relief of inhibition. Pterostilbene; BLF: bamboo leaf flavonoids; Hesperidin; Apigenin; IOP: *Inonotus obliquus* polysaccharide.

The dual antioxidant and autophagy-regulating functions of plant-derived compounds are functionally integrated through crosstalk between key signaling pathways, including Nrf2, AMPK, and MAPK (Figure 4). These pathways act as central “signaling hubs” that coordinate antioxidant defense by inducing antioxidant protein expression to promote ROS scavenging. They also maintain autophagic homeostasis by regulating autophagic processes and autophagic flux. This intricate pathway crosstalk not only unifies the dual biological functions of plant-derived compounds but also amplifies their combined effects to exert a robust synergistic skin protection effect.

#### 5.2.1. Nrf2 Pathway

The Nrf2 pathway is the core hub linking antioxidation and autophagy. It activates downstream antioxidant target genes (HO-1, NQO-1). This directly enhances cellular antioxidant capacity. It also upregulates autophagy-related proteins or promotes autophagosome function. This connects “reducing oxidative stress” and “clearing damaged substances”. A synergistic skin protective network is thus constructed.

Pterostilbene acts on HaCaT cells. It activates Nrf2 via ERK, AMPK, and ROS pathways. It promotes HO-1 and NQO-1 (antioxidant proteins) expression. It enhances autophagy (LC3-II accumulation, AVO formation). This relies on Nrf2 activation to promote melanosome degradation. Nrf2 knockdown attenuates its antioxidant and autophagic activities [72]. Ellagic acid (EA) acts on UVA-exposed HaCaT cells. It activates Nrf2 to inhibit UVA-induced ROS and α-melanocyte-stimulating hormone (α-MSH). It induces autophagy in B16F10 cells. This promotes melanosome clearance for skin lightening. A “melanin synthesis inhibition + degradation acceleration” synergy forms [102]. γ-Mangostin (GM) regulates the Keap1/Nrf2 pathway. It activates antioxidant function and Nrf2 nuclear translocation. It upregulates HO-1 and other antioxidant proteins. It inhibits mTOR to activate autophagy. This reduces UVB-induced cellular senescence and collagen degradation. Blocking autophagy reverses its anti-skin aging effect. This confirms Nrf2-mediated dual function synergy [73]. Apigenin acts on H_2_O_2_-exposed B16F10 melanocytes. It activates the Nrf2 pathway to alleviate oxidative stress. It inhibits the PI3K/Akt/mTOR pathway. It also activates PINK1/PARKIN-mediated mitophagy. The two mechanisms synergistically repair oxidative damage. They also inhibit premature cellular senescence [87]. Resveratrol activates the Nrf2-mediated antioxidant pathway. It upregulates antioxidant-related gene expression. It activates autophagic and lysosomal functions. This promotes melanosome degradation. Nrf2 connects antioxidation and the autophagy–lysosomal system. It synergistically improves skin hyperpigmentation [85].

#### 5.2.2. AMPK Pathway

The AMPK pathway is a core node for energy sensing and metabolic regulation. It integrates antioxidation and autophagy to exert synergistic effects. On one hand, it directly regulates autophagy-related targets. This enhances autophagic flux in skin cells. On the other hand, it indirectly regulates antioxidant pathways (e.g., Nrf2). This promotes antioxidant enzyme expression. It integrates “oxidative stress alleviation” and “cellular homeostasis maintenance”. It adapts to anti-photoaging and anti-pollution skincare scenarios.

*Inonotus obliquus* polysaccharide (IOP) activates AMPK. This enhances autophagy and alleviates UVB-induced autophagy inhibition. It also activates the Nrf2/HO-1 pathway. This promotes HO-1, SOD, and CAT (antioxidant proteins) expression [103]. It synergistically inhibits cellular senescence and apoptosis. This exerts anti-skin photoaging effects. Rose extract (RE) acts on a Benzo[a]pyrene (BAP) + UV-A-induced pollution stress model. RE activates the AMPK pathway. It upregulates Nrf2 and Lysosome-Associated Membrane Protein 2A (LAMP2A). This jointly alleviates keratinocyte senescence and proliferation arrest. An AMPK inhibitor blocks these dual effects. This confirms AMPK’s integrative hub role [71].

#### 5.2.3. MAPK Pathway

The MAPK pathway includes ERK, JNK, and p38 subfamilies. It acts as a stress response hub for skin cells against exogenous damage. It regulates subfamily phosphorylation status. This achieves bidirectional crosstalk between oxidative damage response and autophagic homeostasis. Plant-derived compounds inhibit overactivated MAPK subfamilies. This reduces oxidative damage and restores autophagic flux. They synergistically alleviate oxidative stress-induced skin aging and barrier damage.

In the AAPH-induced HaCaT cell senescence model, MAPK subfamilies (p38, JNK, ERK) are activated. This leads to oxidative damage and autophagy inhibition [76]. Bamboo leaf flavonoids (BLF) exert dose-dependent effects. They inhibit p38 phosphorylation. They upregulate Beclin-1 and the LC3-II/LC3-I ratio. This activates autophagy. BLF restores SOD, CAT, and Glutathione peroxidase (GPx) activities. They reduce MDA and oxidative damage marker levels. Dehydrocorydaline (p38 activator) reverses BLF’s effects. SB203580 (specific p38 inhibitor) enhances BLF’s effects. These results confirm p38 MAPK as BLF’s specific target. It links oxidative damage alleviation–autophagy activation [76]. Hesperidin acts on PM_2.5_-exposed skin cells. It inhibits excessive phosphorylation of p-ERK, p-JNK, and p-p38. It downregulates abnormal Beclin-1 and LC3-II expression. It restores autophagic flux. It reduces ROS generation and MDA accumulation. This alleviates oxidative damage. Combining hesperidin with MAPK inhibitors (U0126/SP600125/SB203580) enhances protection. This confirms MAPK as hesperidin’s core target [77]. Ginsenoside Rg3 acts in a skin wound healing model. Wound-site oxidative stress activates MAPK and NF-κB pathways. This causes excessive inflammatory infiltration and autophagy inhibition. Rg3 inhibits excessive phosphorylation of these pathways. It reduces ROS generation and lipid peroxidation. It relieves pathway-mediated autophagy inhibition. It promotes Beclin-1/LC3-mediated autophagy activation. This facilitates clearance of wound damage components [104].

### 5.3. Synergistic Skin Protection Effects of Dual-Functional Plant-Derived Bioactives

Plant-derived compounds’ antioxidation–autophagy synergy amplifies skin protection. This forms a “functional complementarity dynamic cycle”. Antioxidation reduces autophagy substrate generation. These substrates include oxidatively damaged proteins and excessive ROS. This lowers cellular damage burden at the source. Autophagy enhances antioxidant efficiency by clearing oxidative products. These products include damaged organelles and abnormal melanosomes. This avoids further damage accumulation. The two synergize in skin lightening, anti-inflammation, and anti-aging. They demonstrate more comprehensive skin protection value.

#### 5.3.1. Skin Lightening Synergy

The synergistic skin lightening effect of dual-functional plant-derived bioactives relies on bidirectional crosstalk between antioxidant and autophagic pathways. This crosstalk integrates two key processes: antioxidant-mediated inhibition of melanin synthesis and autophagy-driven clearance of melanin. Antioxidant activity scavenges ROS to reduce melanin production and downregulates melanogenic factors such as MITF and tyrosinase. Meanwhile, autophagy promotes the degradation of mature melanosomes by enhancing lysosomal degradation function, thereby accelerating melanin clearance. Collectively, these two interconnected mechanisms synergistically reduce epidermal melanin deposition.

*Piper Betle* leaf ethanol extract (PBLE) activates autophagy. It upregulates LC3-II, Atg5, Beclin-1, and promotes melanosome degradation. Its ethyl acetate fraction (PBLA) exhibits strong antioxidant activity. It scavenges DPPH, ABTS, and NO free radicals. In UVB-induced mice, PBLE dose-dependently reduces melanin deposition [105]. Tea leaf-derived nanovesicles (TLNVs) scavenge ABTS and 2-phenyl-4,4,5,5-tetramethylimidazoline-1-oxyl-3-oxide (PTIO) free radicals. This reduces ROS-mediated melanin synthesis. TLNVs activate autophagy by upregulating LC3B and downregulating p62. This promotes melanosome degradation. They also target the miR-828b/MYB4 axis. This synergistically downregulates MITF and tyrosinase [84].

#### 5.3.2. Anti-Inflammatory and Barrier Repair Synergy

This synergy relies on a progressive functional relationship. It combines antioxidation-mediated inflammation initiation blocking. It also includes autophagy-driven inflammatory mediator clearance. Antioxidation reduces ROS generation. This blocks activation of NF-κB and other inflammatory signals. Autophagy clears pro-inflammatory factors and damaged cellular components. It alleviates inflammatory damage to the skin barrier. It also promotes expression of barrier-related proteins. This adapts to inflammation-associated skin problems.

7-Methylsulfonylheptyl isothiocyanate (7-MSI) is a plant-derived active component. It is isolated from Brassicaceae (cruciferous) plants. 7-MSI reduces LPS-induced ROS generation. It inhibits the NF-κB/NOD-like receptor pyrin domain-containing 3 (NLRP3) inflammasome pathway. It also activates autophagy in relevant cells. These actions synergistically inhibit pro-inflammatory factor expression. Autophagy gene silencing reverses its anti-inflammatory effect. This confirms the “antioxidation–autophagy” synergistic mechanism [106].

#### 5.3.3. Anti-Aging and Barrier Repair Synergy

This synergy combines two key guarantees for skin health. One is antioxidation-mediated reduction in damage input. The other is autophagy-driven repair of damaged structures. Antioxidation alleviates UVB-induced endoplasmic reticulum stress. It also mitigates UVB-induced mitochondrial dysfunction. This reduces collagen degradation and senescence marker expression. Autophagy clears damaged mitochondria and senescent components. It maintains fibroblast viability and epidermal barrier integrity. This delays skin aging and promotes barrier repair.

Rosmarinic acid (RA) acts on UVB-induced human dermal fibroblasts (HDFs). It downregulates glucose-regulated protein 78 (GRP78) and C/EBP homologous protein (CHOP) [17]. This alleviates endoplasmic reticulum stress. RA upregulates PINK1/PARKIN to enhance mitophagy. It reduces epidermal thickness and inflammatory infiltration in Balb/C mice. RA synergistically exerts anti-photoaging and barrier protection effects [17].

*Myrothamnus flabellifolia*-*Coffea arabica* seed extract (MflCas) acts on HDFs. It reduces protein carbonylation levels in these cells. It upregulates LC3B and downregulates mTOR. It also downregulates autophagy-related protein 7 (Atg7). This activates autophagy. It alleviates protein oxidative damage and clears senescence-related products. MflCas synergistically exerts anti-aging effects [88].

#### 5.3.4. Damage Prevention and Clearance Synergy

This synergy constructs a skin protective loop. It combines the blocking of the occurrence of antioxidation-mediated damage. It also includes autophagy-driven existing damage clearance. Antioxidation inhibits UVA and pollutant-induced ROS generation. This reduces primary damage (denatured proteins, DNA damage). Autophagy enhances the degradation of photo-damaged denatured proteins. It also degrades lipofuscin in skin cells. This avoids secondary damage from damage accumulation. Secondary damage includes cellular senescence and dysfunction. It adapts to anti-photoaging and anti-pollution scenarios.

Handelin acts on UVA-induced human dermal fibroblasts (HDFs). It inhibits ROS generation to reduce photo-damaged denatured proteins. It upregulates LC3-II and heat shock protein 70 (HSP70) to enhance autophagy. Enhanced autophagy accelerates denatured protein clearance. It synergistically reduces senescence markers (p21, SA-β-gal). This achieves anti-photoaging via “new damage prevention + old damage clearance” [89].

In summary, plant-derived compounds have dual antioxidation–autophagy functions. They follow a “material basis-pathway regulation-effect manifestation” logic. This constructs an efficient skin protection network. These ingredients compensate for single antioxidation limitations. Single antioxidation only prevents new damage. They also overcome single autophagy regulation deficiencies. Single autophagy lacks damage source blocking. These advantages support precise natural skincare ingredient development.

## 6. Translational Application of Plant-Derived Bioactive Compounds in Skin Protection: Formulation Development and Clinical Efficacy Validation

**Figure 5:**
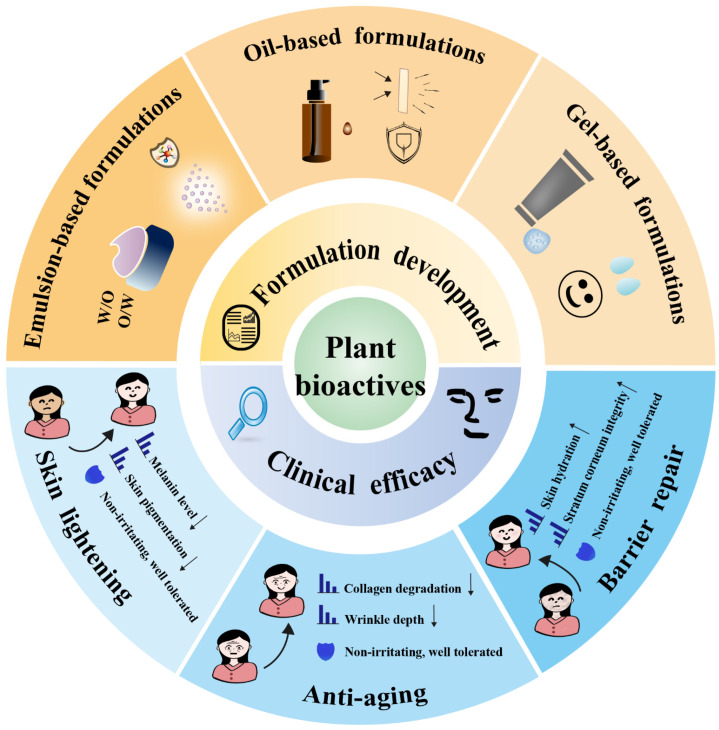
Plant-Derived Compounds: formulation development and clinical efficacy validation for skin protection. Arrows indicate an increase (↑) or decrease (↓) in the corresponding indicator.

Plant-derived bioactive compounds have shown considerable potential for skin protection based on their antioxidant and autophagy-regulating properties. To translate this potential into practical applications, it is essential to focus on two key areas (Figure 5). First, the development of suitable formulations to optimize stability, skin permeability, and synergistic effects. Second, clinical evaluations verify the preliminary efficacy of these compounds, which provide fundamental support for their practical applications in skin lightening, anti-aging, and skin barrier repair.

### 6.1. Formulation Development for Skin-Protective Plant-Derived Compounds

Functional preparation development is a key part of translational research. Its core logic is “ingredient properties–dosage form characteristics–skin protection requirements” matching. Select dosage forms (emulsions, oils, gels) based on ingredient solubility (water-soluble or liposoluble). Also consider ingredient stability and transdermal potential. Use loading technologies and emulsification optimization to enhance efficacy release and improve biocompatibility and effective delivery, toward safe, stable, and efficient skin protection.

#### 6.1.1. Emulsion-Based Formulations (Creams/Lotions)

Emulsion-based formulations utilize the synergistic interaction between oil and water phases, making them excellent carriers for plant polyphenols, flavonoids and essential oils. They can be classified into water-in-oil (W/O) and oil-in-water (O/W) systems [107,108]. The key advantage of emulsion formulations lies in their ability to utilize a single or double interface barrier to protect water-soluble and lipid-soluble active ingredients from degradation, whilst enhancing ingredient dispersion and skin delivery efficiency. They offer a wide range of skincare benefits.

Through rational design and technical development, the performance of emulsions can be further optimized to improve the skin-related effects of plant-derived active ingredients.

Nanoemulsions can enhance the stability of water-soluble active ingredients. A W/O nanoemulsion with macadamia oil and tea tree essential oil as the oil phase and vitamin C as the aqueous phase exhibits superior antioxidant activity compared to a single vitamin C solution. This nanoemulsion reduces the oxidative degradation of active ingredients through a dual interfacial barrier. It also improves the dispersibility of plant-derived fat-soluble components, providing a technical solution for the synergistic cutaneous delivery of unstable active ingredients and plant active ingredients in emulsions [107].

For the multifunctional synergistic design of plant extracts, a study constructed an O/W sunscreen emulsion with *Calendula arvensis* extract as the core, grapeseed oil-beeswax as the oil phase, and a composite emulsifier as the stabilizer. The formulation matches the solubility and release requirements of plant active ingredients. By optimizing the concentration of the added extract, it achieves UVB protection and antioxidant effects. Meanwhile, the emulsion inhibits the activities of tyrosinase and elastase, realizing anti-pigmentation and anti-skin aging efficacies [108].

In addition, the efficient cutaneous delivery of active ingredients can be enhanced by constructing a multiple emulsion system. A “water-oil-water” three-layer structure was established with *Rosmarinus officinalis* L., *Avena sativa* L., and *Linum usitatissimum* L. extracts as the core. Through dual physical barriers, it synchronously protects water-soluble and fat-soluble active ingredients from environmental degradation. Natural antibacterial activity can also be achieved by virtue of the phenolic acids in the extracts themselves. Validated by the EpiDerm™ 3D skin model and human patch tests, this W/O/W multiple emulsion has excellent skin compatibility without irritating or phototoxic reactions. It provides a more stable guarantee for the synergistic delivery of multiple components [109].

#### 6.1.2. Oil-Based Formulations (Essential Oils/Skincare Oils)

Oil-based formulations are commonly used as carriers for fat-soluble plant ingredients such as essential oils and cold-pressed seed oils [110,111,112]. The key advantage of oil-based formulations lies in the fact that the oil-based system enhances the diffusion and penetration of bioactive ingredients [113]. They are suitable for addressing skincare needs such as anti-inflammatory and anti-aging benefits.

Through rational formulation design and component compounding, the performance of oil-based formulations can be further optimized to improve the skin-related effects of plant-derived active ingredients.

For cold-pressed seed oils, the unsaturated fatty acids they contain can synergize with other bioactive components to enhance antioxidant effects. Cold-pressed rosehip oil (*Rosa canina* L.) is rich in polyunsaturated fatty acids, bioactive carotenoids, and phenolic compounds. These components exert synergistic antioxidant effects by scavenging ROS. They also possess anti-skin aging efficacy, including promoting collagen synthesis, reducing wrinkles, and improving skin texture [112].

For plant essential oils, their bioactive components can exert targeted anti-inflammatory and anti-aging effects. *Eucalyptus citriodora* essential oil contains 54.9% citronellal and 25.4% citronellol, which are its major bioactive constituents. At a concentration of 10 μg/mL, this essential oil significantly inhibits the release of superoxide anions and the secretion of elastase from human neutrophils. Superoxide anions and elastase are core biomarkers of inflammatory responses. By targeting these key molecules, the essential oil not only exerts anti-inflammatory effects but also achieves anti-aging efficacy by alleviating oxidative damage and protecting the integrity of skin structure [114].

Furthermore, oil-based formulations can enhance skincare benefits through the synergistic action of various plant-derived active ingredients. The lipophilic nature of these oil-based systems facilitates the spreading and penetration of bioactive plant components on the skin surface. This allows the compounded ingredients to act synchronously on the skin, further strengthening the synergistic effect. Clove oil, with eugenol as the core, is compounded with terpenoid components such as trans-caryophyllene and α-caryophyllene, which can enhance the antioxidant capacity of plant bioactive components. Citronella oil integrates aldehyde and alcohol components, including citronellal, citronellol, and geraniol, thereby improving antioxidant skincare efficacy [113].

#### 6.1.3. Gel-Based Formulations (Gels/Hydrogels)

Gel formulations are well-suited for water-soluble plant-based ingredients. These formulations have a high-water content, provide excellent moisturization and are gentle on the skin. At the same time, they harness the anti-inflammatory, soothing and antibacterial properties of plant-derived active ingredients [115,116]. They are therefore suitable for skin types with low tolerance. They offer a wide range of skincare benefits and can meet the needs of various skincare scenarios [117,118].

Through rational carrier optimization and formulation design, the performance of plant-derived gel formulations can be further optimized to enhance efficacy for targeted skin care needs.

For hydrophobic plant ingredients, nano-carrier gel composites effectively address instability and solubility issues. Quercetin-loaded chitosan lactate nanoparticle-xanthan gum gel resolves quercetin’s poor solubility and oxidation problems, with a DPPH IC_50_ value significantly lower than the blank hydrogel. This gel also exhibits antibacterial activity against skin-related pathogens, making it suitable for skin with microecological disorders [119].

For targeted skin repair, responsive gel systems are designed to achieve site-specific effects. A thermosensitive gel composed of Poloxamer 407/chitosan/hyaluronic acid (HA) is used to load the plant-derived bioactive component taxifolin. This gel activates MAPK pathway-mediated autophagy, promotes Vascular Endothelial Growth Factor (VEGF) and Pan-Keratin expression, and accelerates skin defect repair [120].

In summary, functional cosmetic formulation development for plant-derived ingredients centers on matching the properties of ingredients, the characteristics of dosage forms, and specific skincare needs. These dosage forms, which are tailored to diverse skincare needs through targeted technological design, provide reliable support for the application of plant-derived bioactive compounds. However, most current formulations focus on optimizing basic properties or targeting skin surface/type-specific needs, and further advancement is still needed for precise delivery to deep skin targets or subcellular organelles.

### 6.2. Clinical Efficacy Validation for Skin-Protective Plant-Derived Compounds

Human clinical trials extend the translational research of plant-derived skin protectants from the laboratory to the clinical application stage. The experimental design is centered on addressing the practical demands of skin whitening, anti-aging, and barrier repair. It assesses the efficacy of active ingredients through metrics including melanin index, wrinkle depth, and skin hydration level. These data provide a reference for subsequent translational studies.

#### 6.2.1. Skin Lightening and Pigment Regulation

Clinical trials have confirmed that plant-derived ingredients exhibit skin whitening and anti-pigmentation effects [121]. Their mechanisms of action involve two key pathways: one involves inhibiting melanin synthesis or melanin transfer, and the other reduces melanin production by scavenging ROS induced by UV radiation. Clinical evaluation typically takes into account melanin content, the number of pigmentation lesions, and skin tone uniformity.

Studies have shown that 1% *Paeonia albiflora* root extract can inhibit pathways related to advanced glycation end products (AGEs). A split-face study enrolled 17 healthy participants aged 40–62 years. After two weeks, cheek melanin content was significantly reduced (*p* < 0.01). VISIA imaging revealed decreased spots and improved skin tone uniformity, with no local skin irritation observed throughout the study [122]. Creams containing Burmese Thanaka bark extract (*Hesperethusa crenulata*) exert mild skin-lightening effects. Their mechanisms include antioxidant activity and mild tyrosinase inhibition. In a 30-day daily application study involving 20 healthy volunteers, Antera 3D imaging demonstrated positive outcomes. These included a downward trend in the facial pigmentation index (baseline: 30.0 ± 12.12 vs. day 30: 22.4 ± 12.45), an approximate 19.4% reduction in average redness (hemoglobin-related), as well as improvements in skin wrinkles and other indicators. No skin dullness or exacerbated pigmentation was observed during the study, indicating good safety [123]. A study involving 20 female subjects aged 35–60 years with “sensitive + photoaged skin” evaluated a composite formulation containing plant extracts. After 56 days of daily topical application, facial hyperpigmented spots showed an average 14% reduction (*p* < 0.01), and skin luminosity increased by an average 69%. Notably, this improvement did not alter basal skin tone. This formulation exerts synergistic effects of antioxidation, anti-inflammation, and skin barrier repair. It can precisely ameliorate photoaging-related pigmentation while enhancing skin elasticity and reducing wrinkles, thereby demonstrating multifunctional advantages in photodamage repair [124].

#### 6.2.2. Anti-Aging and Barrier Repair

Clinical data demonstrate that plant-derived ingredients exert multi-dimensional efficacy in anti-aging and skin barrier repair [112,125]. Their mechanisms primarily involve three aspects. First, they inhibit the enzymatic activity of collagenase. Second, they promote the synthesis of collagen and filaggrin. Third, they reinforce skin barrier function. Clinical validation typically involves multi-dimensional parameter assessments. These include wrinkle depth, skin elasticity, moisture retention, and barrier function.

Studies have shown that a facial serum utilizes 10% Sacha Inchi oil as its core ingredient. This oil is extracted from the seeds of *Plukenetia volubilis* L. It inhibits neutrophil elastase by 71.96% and collagenase by 66.67%. Its efficacy is superior to that of retinoids. A 48 h occlusive patch test involving 20 subjects revealed no erythema or stinging reactions. It is suitable for anti-aging application on sensitive skin [126]. A cream containing 0.3% Prunus cerasifera leaf extract was tested on 26 healthy volunteers aged 30–50 years. The study confirmed that it possesses significant antioxidant activity and good skin safety. It also demonstrates promising potential for preventing skin aging [127]. Twenty-seven volunteers aged 25–65 years used *Rosa canina* (rosehip oil) daily for 5 weeks. Significant age-stratified effects were observed across the groups. In the young population (25–35 years old), true skin age decreased and skin texture improved. This helps maintain a youthful skin state and delays premature aging. In the population aged 36–65 years, wrinkle depth was reduced. Photoaging-related spots showed an improving trend [112]. A 12-week, randomized, double-blind, placebo-controlled study involved 21 healthy women aged 30–59 years. A cream containing 0.1% *Phaseolus angularis* seed extract (PASE) significantly improved periorbital skin condition. Smoothness depth (R4) was reduced by 18.6%. The arithmetic mean roughness (R5) was reduced by 25.0% (*p* < 0.05). Meanwhile, the cream possesses long-lasting moisturizing efficacy. Skin moisture content remained significantly higher than that of the placebo group 24 h after application. It was well-tolerated in dry skin (48% of subjects) and normal skin (42% of subjects) [128]. A 3% fermented tea extract (FTE) emulsion was tested on 66 volunteers for 28 and 56 days. The results showed that it possesses multiple effects, including moisturization, skin barrier repair, anti-wrinkle, and soothing properties. Notably, efficacy after 56 days of use was superior to that after 28 days. Long-term use yields more significant effects [125]. A 2% composite essential oil blend containing *Lavandula angustifolia*, *Eucalyptus globulus*, *Citrus reticulata*, and *Melaleuca alternifolia* was tested on 40 male subjects for 90 days. The results showed that the product increases stratum corneum water content. It optimizes stratum corneum morphology, significantly regulates sebum secretion (oil control), reduces transepidermal water loss (TEWL), and improves skin barrier function without causing barrier damage. The product was well-tolerated by healthy skin. Leveraging its dual functions of oil control and barrier repair, it is well-suited for both oily skin and the barrier repair of sensitive skin [129].

In summary, the results of existing clinical studies suggest that plant-derived active ingredients possess strong application value in skin whitening, anti-aging, and skin barrier repair. Table 1 summarizes several relevant clinical investigations. Overall, these studies cover diverse formulations and efficacy endpoints, with most focusing on healthy subject populations (a few involving sensitive + photoaged skin [122,124]). Their intervention periods range from short-term assessments to long-term observations lasting up to three months. Relevant clinical indicators mainly focus on parameters such as pigment profiles, wrinkle depth, skin moisture content and transepidermal water loss. Data trends generally indicate that plant-derived active ingredients can improve uneven skin tone, alleviate skin aging symptoms and assist in barrier repair, exerting certain positive effects.

Notably, some high-quality studies have adopted rigorous designs such as randomized, double-blind, placebo-controlled trials and achieved large sample sizes or long intervention periods, providing reliable evidence for clinical translation [125,129]. However, many other studies suffer from inherent limitations. Such limitations include small sample sizes, short intervention durations, and a lack of controlled experimental designs. Despite these drawbacks, the tested formulations generally exhibit favorable skin safety and tolerability. No obvious adverse reactions have been observed in clinical trials. To enhance the reliability of plant-derived active ingredients in clinical skincare, more in-depth validation studies are urgently needed. These studies should use standardized experimental designs and multi-center trials across diverse skin type scenarios. This approach will further promote the practical clinical translation of plant-derived active ingredients.

## 7. Research Challenges and Future Perspectives

With the deepening research on plant-derived bioactive ingredients in skin protection, they have become a research hotspot in the skincare field due to their unique advantages of natural mildness and multi-target synergy. Phased progress has been made in both basic mechanism exploration and formulation technology development. However, in the full chain from basic theory to clinical translation, problems such as insufficient elucidation of specific mechanisms and incomplete translational application still exist.

### 7.1. Mechanistic Gaps and Research Challenges of Antioxidation and Autophagy in Skin Protection

Although research on antioxidation and autophagy has advanced, it offers key theoretical support for skin protection. However, many critical specific mechanistic gaps await being filled.

Classical antioxidant pathways are systematically studied in skin cells, but the regulation of emerging targets lacks full verification. Taking *RAC2* as an example, current studies only confirm its antioxidant effect in the natural senescence model of human dermal fibroblasts [13]. It remains unclear whether it exerts equivalent antioxidant effects in epidermal keratinocytes compared to dermal fibroblasts. Meanwhile, whether the regulatory logic under UV irradiation and PM_2.5_ exposure is consistent with the natural senescence model also awaits confirmation. Resolving these issues is crucial to determining its potential as a broad-spectrum skin-protective target. Regarding *LINC00294*, although isoschaftoside has been shown to significantly downregulate its expression in naturally senescent dermal fibroblasts [13], the specific interactive mechanisms between this long non-coding RNA and its associated RNA-binding proteins and target genes remain largely unelucidated. This represents a key mechanistic gap that needs to be further addressed in current research.

In the field of autophagy regulation, particularly mitophagy, the core “component–autophagy receptor” interaction logic is not clearly defined in skin. For example, the precise mechanism by which rosmarinic acid regulates mitophagy via OPTN and NDP52 receptors remains unclear [17]. How polydatin coordinates inter-organelle communication during mitophagy via the miR-155/TFAM axis is also an unsolved scientific gap [18].

Beyond single-mechanism deficiencies, the temporal order of the synergistic regulation between antioxidation and autophagy lacks experimental evidence [102,104]. It is unclear whether antioxidation repairs mitochondria first to reduce ROS levels, autophagy clears damaged organelles earlier, or both initiate simultaneously. This ambiguity in sequence and synergistic logic directly hinders the optimal application of dual mechanisms in skincare.

### 7.2. Shortcomings and Limitations of Formulation and Clinical Research in Skin Protection

Although progress has been made in formulation design and clinical validation of plant-derived active ingredients, key transformation bottlenecks still exist when applying them to complex real skin environments.

Despite the advancements in enhancing transdermal absorption and achieving basic skin-type-specific improvement through optimized formulations, critical gaps still exist in achieving precise mechanistic targeting for topical applications. Specifically, most current development focuses on fundamental performance improvements [107,113]. However, efforts to target deeper skin action targets (e.g., mitochondria in dermal fibroblasts) and realize subcellular-level efficacy remain challenging. This lack of comprehensive targeting prevents the active ingredients from fully exerting their core effects. It is difficult to precisely deliver active ingredients to sites exerting core functions such as oxidation resistance and autophagy regulation.

At the clinical validation level, existing studies have confirmed the potential of plant-derived actives in skin whitening, anti-aging, and barrier repair through diverse trial designs. However, several structural bottlenecks still hinder their clinical translation. First, long-term stability and efficacy validation remain insufficient. Most clinical studies only capture short-term changes observed over days to weeks, and systematic confirmation of long-term skin homeostasis maintenance (e.g., beyond 100 days) remains insufficient [122,127]. Additionally, an imbalance exists between sample sizes and intervention durations. For instance, some studies achieve relatively large sample sizes, such as the 66 subjects included in fermented tea extract research. However, their intervention duration is limited to 56 days [125]. Conversely, other studies implement extended follow-up periods, such as approximately 90 days of intervention. Unfortunately, their subject numbers only reach 40 cases, which is relatively small [129]. Furthermore, rigorous trial designs are not universally adopted. Although randomized, double-blind, and placebo-controlled protocols have been attempted in certain studies [125,128,129], the majority still rely on single-arm or non-controlled frameworks. This limits the comprehensive validation and broad extrapolation of existing findings. Finally, the study population is limited to healthy skin [125,129]. Most data are derived from healthy subjects, and there is a lack of systematic exploration on heterogeneous skin types such as sensitive skin and severe photoaging of skin. This “idealized” sample makes it difficult to extrapolate conclusions to more challenging real-world repair scenarios.

In summary, research on plant-derived bioactive ingredients in skin protection has laid the core foundation of the “antioxidation–autophagy” dual mechanism. It has also achieved breakthroughs such as stable delivery and enhanced transdermal absorption through formulation optimization. However, in the transition from “basic research” to “precision skincare application”, this field still faces dual challenges of mechanism deepening and translational implementation. These challenges also point out the core direction for future research.

At the mechanism level, it is necessary to focus on addressing key gaps in skin protection research. These gaps include multi-scenario validation of emerging targets in antioxidant pathways and elucidation of upstream and downstream interaction mechanisms of key molecules. They also involve the binding logic of “component–autophagy receptor” in autophagy regulation and the temporal order of antioxidation–autophagy synergy. These efforts will provide theoretical support for the precise regulation of the dual mechanism. At the translational application level, formulation development needs to break through the bottleneck of targeted delivery to deep skin targets and subcellular organelles. Clinical research should expand sample sizes, improve control design, and cover diverse skin types to enhance the clinical credibility and universality of results. Overcoming these challenges will be crucial to promoting the optimal application and releasing the greater value of plant-derived bioactive ingredients in the skincare field.

## 8. Conclusions

This review summarizes the skin-protective effects of plant-derived bioactive compounds. It focuses on antioxidant activity and autophagy regulation. Research constructs a comprehensive “component–pathway–efficacy” logical chain. It integrates classic and emerging antioxidant targets, as well as universal and selective autophagy pathways. More importantly, this study establishes an integrated antioxidant–autophagy synergistic regulatory model. This dual-mechanism framework clarifies the core coordination mechanisms and key pathways of dual-functional plant-derived active ingredients in natural skincare. Research also summarizes advancements in formulation development and clinical efficacy validation. This review provides theoretical references for skin health research driven by plant-derived bioactive compounds. It also offers theoretical support for the application, translation and efficient development of active ingredients.

## Figures and Tables

**Table 1:** Summary of clinical studies on skin whitening, anti-aging and barrier repair.

No.	Compound/ Extract	Model	Sample Size	Study Duration	Study Design	Endpoint	Safety	Key Limitations
1	*Paeonia albiflora* root extract [122].	Healthy participants (with visible facial dullness or pigmentation).	17 subjects (40–62 years)	2 weeks	Randomized, single-blinded, split-face controlled study.	↓ Cheek melanin content (*p* < 0.01); ↓ facial spots (VISIA); ↓ AGEs accumulation (trend); improved skin tone uniformity.	No adverse skin reactions observed.	Small sample size; short intervention period.
2	Thanaka bark extract (*Hesperethusa crenulata*) [123].	Healthy participants.	20 subjects (25–55 years)	30 days	Single-arm, single-blind clinical trial.	↓ Pigmentation index; ↓ melanin content; ↓ average redness (19.4%); ↓ wrinkles/fine lines/pore density; ↑ skin hydration/elasticity; ↓ TEWL.	No local skin irritation was observed.	Small sample size; short intervention period; The study design was single-arm without a control group.
3	Composite plant extract formulation (*Hylocereus undatus*, *Punica granatum* seed, *Physalis angulata*, etc.) [124].	Healthy participants (with sensitive + photoaged skin).	20 female subjects (35–60 years)	56 days	Single-arm, open-label clinical trial.	↓ Facial hyperpigmentation spot melanin (14%, *p* < 0.01); ↑ skin luminosity (69% in responders); no change in baseline skin tone; ↑ skin hydration (31%), ↓ TEWL (20%), ↑ firmness/elasticity (up to 30%), ↓ wrinkle volume (29%), ↓ wrinkle density (7.6%, *p* < 0.01).	No adverse reactions were observed.	Small sample size; Mid-term observation with limited duration; The study design was single-arm without a control group.
4	Sacha inchi oil (*Plukenetia volubilis* L.) [126].	Healthy participants.	20 subjects (mean age 22 years)	48 h	Single-arm, open-label skin irritation test (occlusive patch method).	Inhibits elastase (71.96%), collagenase (66.67%); superior to retinol.	No adverse reactions were observed.	Small sample size; short intervention period.
5	*Prunus cerasifera* leaf extract [127].	Healthy participants.	26 subjects (30–50 years)	48 h	Double-blind, semi-open patch test.	Exhibits stable antioxidant activity; potential to prevent skin aging.	No adverse skin reactions observed.	Small sample size; short intervention period.
6	*Rosa canina* seed oil [112].	Healthy participants.	26 subjects (25–65 years)	5 weeks	Open-label, non-blinded clinical trial.	↓ True skin age (AG1: 33.5→29.6, *p* < 0.05); ↓ wrinkle score (AG4: 37.1→30.2); ↓ skin texture score; improved photoaging-related brown spots and UV spots.	No serious adverse reactions were observed during the study. Only a few subjects had mild erythema or itching.	Small sample size; short intervention period; non-blinded without a control group.
7	*Phaseolus angularis* seed extract [128].	Healthy participants.	21 females (30–59 years)	12 weeks	Single-center, randomized, double-blind, placebo-controlled clinical trial.	↓ Periocular wrinkle depth (R4: 18.6%, *p* < 0.05); ↓ skin arithmetic mean roughness (R5: 25.0%, *p* < 0.05); ↑ 24 h skin hydration (significantly higher vs. placebo).	No adverse skin reactions observed.	Small sample size.
8	Fermented tea extract [125].	Healthy participants.	66 subjects (mean age 39.59 years)	28/56 days	Single-center, randomized, double-blind, placebo-controlled clinical trial.	↑ Skin hydration and elasticity (R2); ↓ TEWL (barrier repair); ↓ wrinkle area/ratio; ↓ erythema area/ratio; all key indicators: 56-day efficacy > 28-day (*p* < 0.001)	No adverse skin reactions observed.	Mid-term observation with limited duration.
9	Composite essential oil (*Lavandula angustifolia*, *Eucalyptus globulus*, *Citrus reticulata*, and *Melaleuca alternifolia* essential oils) [129].	Healthy participants.	40 male subjects (18–28 years)	90 days	Single-center, randomized, double-blind, placebo-controlled clinical trial.	↑ Stratum corneum hydration (SCWC); ↓ TEWL (barrier repair); ↓ superficial sebum content (*p* < 0.001); ↓ sebaceous gland activity (*p* = 0.021); improved stratum corneum morphology (regular granular layer honeycomb pattern, reduced comedone size)	No adverse skin reactions observed.	—

Note: ↑ = increased, ↓ = decreased.

## Data Availability

No new data were created or analyzed in this study. Data sharing is not applicable to this article.

## References

[1] Song S., Li F., Zhao B., Zhou M., Wang X. (2025). Ultraviolet Light Causes Skin Cell Senescence: From Mechanism to Prevention Principle. Adv. Biol..

[2] Papaccio F., D′Arino A., Caputo S., Bellei B. (2022). Focus on the Contribution of Oxidative Stress in Skin Aging. Antioxid.

[3] Shin S.H., Lee Y.H., Rho N.-K., Park K.Y. (2023). Skin Aging from Mechanisms to Interventions: Focusing on Dermal Aging. Front. Physiol..

[4] Markiewicz E., Idowu O.C. (2022). Evaluation of Personalized Skincare Through In-Silico Gene Interactive Networks and Cellular Responses to UVR and Oxidative Stress. Clin. Cosmet. Investig. Dermatol..

[5] He X., Gao X., Guo Y., Xie W. (2024). Research Progress on Bioactive Factors against Skin Aging. Int. J. Mol. Sci..

[6] Lee S.J., Kim J.E., Choi Y.J., Jin Y.J., Roh Y.J., Seol A.Y., Song H.J., Park S.H., Uddin M.S., Lee S.W. (2022). Antioxidative Role of *Hygrophila erecta* (Brum. F.) Hochr. on UV-Induced Photoaging of Dermal Fibroblasts and Melanoma Cells. Antioxidants.

[7] Onishi M., Yamano K., Sato M., Matsuda N., Okamoto K. (2021). Molecular Mechanisms and Physiological Functions of Mitophagy. EMBO J..

[8] Bandyopadhyay A., Selvan S.A., Patial P.K., Pal T. (2025). Plant-based Ingredients in Cosmetic Science: Current Applications, Limitations, and Prospects. Intern. J. Cosmet. Sci..

[9] Nadeeshani Dilhara Gamage D.G., Dharmadasa R.M., Chandana Abeysinghe D., Saman Wijesekara R.G., Prathapasinghe G.A., Someya T. (2022). Global Perspective of Plant-Based Cosmetic Industry and Possible Contribution of Sri Lanka to the Development of Herbal Cosmetics. Evid. Based Complement. Altern. Med..

[10] Kriauciunaite A., Ferolla P. (2025). Skin Care Path to Purchase: Selection, Purchase, Engagement.

[11] Hu X., Chen M., Nawaz J., Duan X. (2024). Regulatory Mechanisms of Natural Active Ingredients and Compounds on Keratinocytes and Fibroblasts in Mitigating Skin Photoaging. Clin. Cosmet. Investig. Dermatol..

[12] Zhao C., Wu S., Wang H. (2025). Medicinal Plant Extracts Targeting UV-Induced Skin Damage: Molecular Mechanisms and Therapeutic Potential. Int. J. Mol. Sci..

[13] Lee Y.H., So B.H., Lee K.S., Kuk M.U., Park J.H., Yoon J.H., Lee Y.J., Kim D.Y., Kim M.S., Kwon H.W. (2024). Identification of Cellular Isoschaftoside-Mediated Anti-Senescence Mechanism in *RAC2* and *LINC00294*. Molecules.

[14] Hartinger R., Singh K., Leverett J., Djabali K. (2024). Enhancing Cellular Homeostasis: Targeted Botanical Compounds Boost Cellular Health Functions in Normal and Premature Aging Fibroblasts. Biomolecules.

[15] Lee K.W., Cho Y.-Y., Kim K.D. (2024). RCHY1 and OPTN: An E3-Ligase and an Autophagy Receptor Required for Melanophagy, Respectively. Autophagy.

[16] Wan J.-Z., Li C.-Q., Li Y.-N., Li A.-Z., Yang Q.-L., Kang M., Cao J.-H., Ran H.-J., Liu Q.-L., Wan Y. (2025). Chikusetsu Saponin IVa Attenuates Aging by Improving Autophagy and Mitophagy. Free Radic. Biol. Med..

[17] Gupta D., Sharma R.R., Rashid H., Bhat A.M., Tanveer M.A., Abdullah S.T. (2023). Rosmarinic Acid Alleviates Ultraviolet-Mediated Skin Aging via Attenuation of Mitochondrial and ER Stress Responses. Exp. Dermatol..

[18] Valenti D., Abbrescia D.I., Marzano F., Ravagnan G., Tullo A., Vacca R.A. (2025). Polydatin Reactivates Mitochondrial Bioenergetics and Mitophagy While Preventing Premature Senescence by Modulating microRNA-155 and Its Direct Targets in Human Fibroblasts with Trisomy 21. Free Radic. Biol. Med..

[19] Sakamoto K., Fujimoto R., Nakagawa S., Kamiyama E., Kanai K., Kawai Y., Kojima H., Hirasawa A., Wakamatsu K., Masutani T. (2023). Juniper Berry Extract Containing Anthricin and Yatein Suppresses Lipofuscin Accumulation in Human Epidermal Keratinocytes through Proteasome Activation, Increases Brightness and Decreases Spots in Human Skin. Int. J. Cosmet. Sci..

[20] Yoon J.H., Park S.H., Yoon S.E., Hong S.Y., Lee J.B., Lee J., Cho J.Y. (2023). Hydrangea Serrata Hot Water Extract and Its Major Ingredient Hydrangenol Improve Skin Moisturization and Wrinkle Conditions via AP-1 and Akt/PI3K Pathway Upregulation. Nutrients.

[21] Reynolds W.J., Bowman A., Hanson P.S., Critchley A., Griffiths B., Chavan B., Birch-Machin M.A. (2021). Adaptive Responses to Air Pollution in Human Dermal Fibroblasts and Their Potential Roles in Aging. FASEB BioAdv..

[22] Ge G., Wang Y., Xu Y., Pu W., Tan Y., Liu P., Ding H., Lu Y.-M., Wang J., Liu W. (2023). Induced Skin Aging by Blue-Light Irradiation in Human Skin Fibroblasts via TGF-β, JNK and EGFR Pathways. J. Dermatol. Sci..

[23] Ferrara F., Yan X., Pecorelli A., Guiotto A., Colella S., Pasqui A., Lynch S., Ivarsson J., Anderias S., Choudhary H. (2024). Combined Exposure to UV and PM Affect Skin Oxinflammatory Responses and It Is Prevented by Antioxidant Mix Topical Application: Evidences from Clinical Study. J. Cosmet. Dermatol..

[24] Wang C., Ali I., Wang D., Hong T., Zhang J., Li C., Yang W. (2021). Polyphenols Separated from Enteromorpha Clathrata by One-Dimensional Coupled with Inner-Recycling High-Speed Counter-Current Chromatography and Their Antioxidant Activities. Eur. Food Res. Technol..

[25] Ma C.-M., Zhao J.-R., Wu F.-F., Zhang Q., Zhao X.-H. (2022). The Non-Covalent Interacting Forces and Scavenging Activities to Three Free Radicals Involved in the Caseinate–Flavonol (Kaempferol and Quercetin) Complexes. Food Meas..

[26] Lu X.-Q., Li J., Wang B., Qin S. (2024). Computational Insights into the Radical Scavenging Activity and Xanthine Oxidase Inhibition of the Five Anthocyanins Derived from Grape Skin. Antioxidants.

[27] Lu X.-Q., Ren P., Qin S., Li J., Zhao X.-N. (2025). Antioxidant Potential of Four Flavonoids from *Scutellaria baicalensis*: A Computational Investigation on Radical Scavenging Activity and Enzyme Inhibition. New J. Chem..

[28] Wang W., Luo J.-Q., Lv B.-Y., Dong Z.-W., Zhai C.-S., Hu Y.-H., Jin Z., Du D., Tang Y.-Z. (2024). Herbacetin Attenuates Oxidative Stress via Activating Nrf2/HO-1 Signaling Pathway in RAW 264.7 Cells and Caenorhabditis Elegans. J. Food Biochem..

[29] Zhang K., Huang Z., Li J. (2025). Probing the Mechanism of Antioxidant and Oxidative Stability Properties of Gallic Acid and Its Esters in Natural Esters: Computational Investigations and Experiments. Renew. Energy.

[30] Gogoi N.G., Rahman A., Dutta P., Saikia J., Baruah A., Handique J.G. (2024). Design, Synthesis, Biological Evaluation and in Silico Studies of Curcumin Pyrrole Conjugates. Chem. Biodivers..

[31] Zhu G., Ye D., Chen X., Wu Y., Yang Z., Mai Y., Liao B., Chen J. (2023). Lignin-Derived Polyphenols with Enhanced Antioxidant Activities by Chemical Demethylation and Their Structure-Activity Relationship. Int. J. Biol. Macromol..

[32] Quiles J., Cabrera M., Jones J., Tsapekos M., Caturla N. (2022). In Vitro Determination of the Skin Anti-Aging Potential of Four-Component Plant-Based Ingredient. Molecules.

[33] Seo J., Lee U., Seo S., Wibowo A.E., Pongtuluran O.B., Lee K., Han S.B., Cho S. (2022). Anti-Inflammatory and Antioxidant Activities of Methanol Extract of *Piper Betle* Linn. (*Piper Betle* L.) Leaves and Stems by Inhibiting NF-κB/MAPK/Nrf2 Signaling Pathways in RAW 264.7 Macrophages. Biomed. Pharmacother..

[34] Moreau M., Naiken T., Bru G., Marteau C., Canaple L., Gourguillon L., Leblanc E., Oger E., Le Mestr A., Mantelin J. (2023). The Impact of PSR^TM^ (Plant Small RNA Technology), Tea Extract, and Its Principal Components on Mitochondrial Function and Antioxidant Properties in Skin Cells. Cosmetics.

[35] Fan Z., Zhou Y., Gan B., Li Y., Chen H., Peng X., Zhou Y. (2023). Collagen-EGCG Combination Synergistically Prevents UVB-Induced Skin Photoaging in Nude Mice. Macromol. Biosci..

[36] He X.-L.-S., Wang N., Teng X., Wang N.-N., Xie Z.-Y., Dong Y.-J., Lin M.-Q., Zhang Z.-H., Rong M., Chen Y.-G. (2024). *Dendrobium Officinale* Flowers’ Topical Extracts Improve Skin Oxidative Stress and Aging. J. Cosmet. Dermatol..

[37] Ali A., Kim E.H., Lee J.-H., Leem K.-H., Seong S., Kim W. (2021). Processed *Scutellaria Baicalensis* Georgi Extract Alleviates LPS-Induced Inflammatory and Oxidative Stress through a Crosstalk between NF-κB and KEAP1/NRF2 Signaling in Macrophage Cells. Appl. Sci..

[38] Lei X. (2025). Mechanisms and Therapeutic Roles of Medicinal Plants in Skin Photoaging. Clin. Cosmet. Investig. Dermatol..

[39] Merecz-Sadowska A., Sadowski A., Zielińska-Bliźniewska H., Zajdel K., Zajdel R. (2025). Network Pharmacology acs a Tool to Investigate the Antioxidant and Anti-Inflammatory Potential of Plant Secondary Metabolites—A Review and Perspectives. Int. J. Mol. Sci..

[40] Sebghatollahi Z., Yogesh R., Mahato N., Kumar V., Mohanta Y.K., Baek K.-H., Mishra A.K. (2025). Signaling Pathways in Oxidative Stress-Induced Neurodegenerative Diseases: A Review of Phytochemical Therapeutic Interventions. Antioxidants.

[41] Kim E., Kim S.-Y., Song J., Jang J., Seo H.H., Lee J.H., Moh S.H. (2025). Skin Recovery by *Lavandula angustifolia* Leaf Callus Extract: Redox Control of Nrf2 Signaling. Free Radic. Biol. Med..

[42] Izumi Y., Kataoka H., Takada-Takatori Y., Koyama Y., Irie K., Akaike A., Kume T. (2023). Isolation and Purification of Harpagogenin as an Nrf2–ARE Activator from the Tubers of Chinese Artichoke (Stachys Sieboldii). Biol. Pharm. Bull..

[43] Sajeeda A., Bhat A.M., Gorke S., Wani I.A., Sidiqui A., Ahmed Z., Sheikh T.A. (2024). Naringenin, a Flavanone Constituent from Sea Buckthorn Pulp Extract, Prevents Ultraviolet (UV)-B Radiation-Induced Skin Damage via Alleviation of Impaired Mitochondrial Dynamics Mediated Inflammation in Human Dermal Fibroblasts and Balb/c Mice Models. J. Photochem. Photobiol. B Biol..

[44] Kraokaew P., Manohong P., Prasertsuksri P., Jattujan P., Niamnont N., Tamtin M., Sobhon P., Meemon K. (2022). Ethyl Acetate Extract of Marine Algae, Halymenia Durvillei, Provides Photoprotection against UV-Exposure in L929 and HaCaT Cells. Mar. Drugs.

[45] Kim D., Hwang I., Ku B., Choi E.-M. (2021). Antioxidant and Skin Anti-Aging Effects of the Aqueous Ethanol Extract of Ginkgo Biloba Leaf: An in Vitro Study Using HaCaT Keratinocytes. Toxicol. Environ. Health Sci..

[46] Zhu S., Qin W., Liu T., Ma H., Hu C., Yue X., Yan Y., Lv Y., Wang Z., Zhao Z. (2022). Modified Qing’e Formula Protects against UV-Induced Skin Oxidative Damage via the Activation of Nrf2/ARE Defensive Pathway. Front. Pharmacol..

[47] Liu H.-M., Cheng M.-Y., Xun M.-H., Zhao Z.-W., Zhang Y., Tang W., Cheng J., Ni J., Wang W. (2023). Possible Mechanisms of Oxidative Stress-Induced Skin Cellular Senescence, Inflammation, and Cancer and the Therapeutic Potential of Plant Polyphenols. Int. J. Mol. Sci..

[48] Cui B., Wang Y., Jin J., Yang Z., Guo R., Li X., Yang L., Li Z. (2022). Resveratrol Treats UVB-Induced Photoaging by Anti-MMP Expression, through Anti-Inflammatory, Antioxidant, and Antiapoptotic Properties, and Treats Photoaging by Upregulating VEGF-B Expression. Oxidative Med. Cell. Longev..

[49] Yoo T.-K., Jeong W.T., Kim J.G., Ji H.S., Ahn M.-A., Chung J.-W., Lim H.B., Hyun T.K. (2021). UPLC-ESI-Q-TOF-MS-Based Metabolite Profiling, Antioxidant and Anti-Inflammatory Properties of Different Organ Extracts of *Abeliophyllum Distichum*. Antioxidants.

[50] Fang M., Lee H.-M., Oh S., Zheng S., Bellere A.D., Kim M., Choi J., Kim M., Yu D., Yi T.-H. (2022). Rosa Davurica Inhibits Skin Photoaging via Regulating MAPK/AP-1, NF-κB, and Nrf2/HO-1 Signaling in UVB-Irradiated HaCaTs. Photochem. Photobiol. Sci..

[51] Lee H., Park C., Kwon D.H., Hwangbo H., Kim S.Y., Kim M.Y., Ji S.Y., Kim D.H., Jeong J.-W., Kim G.-Y. (2021). Schisandrae Fructus Ethanol Extract Attenuates Particulate Matter 2.5-Induced Inflammatory and Oxidative Responses by Blocking the Activation of the ROS-Dependent NF-κB Signaling Pathway. Nutr. Res. Pract..

[52] Bae I.A., Ha J.W., Boo Y.C. (2023). Chlorogenic Acid, a Component of *Oenanthe Javanica* (Blume) DC., Attenuates Oxidative Damage and Prostaglandin E2 Production Due to Particulate Matter 10 in HaCaT Keratinocytes. Cosmetics.

[53] Kim M., Ha L.-K., Oh S., Fang M., Zheng S., Bellere A.D., Jeong J., Yi T.-H. (2022). Antiphotoaging Effects of Damiana (Turnera Diffusa) Leaves Extract via Regulation AP-1 and Nrf2/ARE Signaling Pathways. Plants.

[54] Lee M., Kim D., Park M.-R., Kim S., Kim J.-L., Kim O.-K., Lee J. (2024). Skin Protective Effect of Indian Gooseberry and Barley Sprout Complex on Skin Dryness, Wrinkles, and Melanogenesis by Cell Models. Nutr. Res. Pract..

[55] Lee S., Oh S., Zheng Q., Zheng S., Kim M., Park S., Choi W., Yin C.S., Yi T.-H. (2024). Photoprotective Effects of *Lithospermum Erythrorhizon* and *Pueraria Lobata* Extracts on UVB-Induced Photoaging: A Study on Skin Barrier Protection. Photodermatol. Photoimmunol. Photomed..

[56] Wang K., Hu X., Xie X.-L., Huang M., Wang D., Yu F.-L. (2024). Phytocosmetic Potential of *Blumea balsamifera* Oil in Mitigating UV-Induced Photoaging: Evidence from Cellular and Mouse Models. J. Ethnopharmacol..

[57] Niu B., An X., Chen Y., He T., Zhan X., Zhu X., Ping F., Zhang W., Zhou J. (2025). *Nigella Sativa* L. Seed Extract Alleviates Oxidative Stress-Induced Cellular Senescence and Dysfunction in Melanocytes. Chin. J. Nat. Med..

[58] Wang L., Yang F., Fu X., Kim Y.-S., Gao X., Jeon Y.-J. (2025). Cosmetic Potential of Agar Oligosaccharides: Anti-Melanogenesis and Photoprotective Effects. Algal Res..

[59] Chen J., Yin Z., Yu N., Ou S., Wang X., Li H., Zhu H. (2024). Tanshinone Alleviates UVA-Induced Melanogenesis in Melanocytes via the Nrf2-Regulated Antioxidant Defense Signaling Pathway. Curr. Mol. Med..

[60] Luangpraditkun K., Kasemkiatsakul P., Sangnim T., Yammen S., Pajoubpong J., Vongsak B. (2025). Anti-Senescence and Anti-Photoaging Activities of Mangosteen Pericarp Extract on UVA-Induced Fibroblasts. Cosmetics.

[61] Zhou Y., He L., Zhang N., Ma L., Yao L. (2022). Photoprotective Effect of Artemisia Sieversiana Ehrhart Essential Oil Against UVB-Induced Photoaging in Mice. Photochem. Photobiol..

[62] Jia Y., Mao Q., Yang J., Du N., Zhu Y., Min W. (2023). (-)-Epigallocatechin-3-Gallate Protects Human Skin Fibroblasts from Ultraviolet a Induced Photoaging. Clin. Cosmet. Investig. Dermatol..

[63] Qu L., Wang F., Chen Y. (2023). Protective Effect and Mechanism Research of *Phyllanthus Emblica*
*Linn*. Fruit Extract on UV-Induced Photodamage in Keratinocytes. Photochem. Photobiol. Sci..

[64] Jung S.-W., Park G.H., Kim E., Yoo K.M., Kim H.W., Lee J.S., Chang M.Y., Shin K.-O., Park K., Choi E.H. (2022). Rosmarinic Acid, as an NHE1 Activator, Decreases Skin Surface pH and Improves the Skin Barrier Function. Int. J. Mol. Sci..

[65] Wang X., Li X., Jiang X., Xiang F., Lai Y., Xiang G. (2023). Efficacy Evaluation of *Chlorella Pyrenoidosa* Extracts on Cytotoxicity Induced by Atmospheric Particulate Matter 2.5 Exposure Using Skin Cell Lines and Zebrafish Models. Cosmetics.

[66] Kong L., Li S., Fu Y., Cai Q., Du X., Liang J., Ma T. (2025). Mitophagy in Relation to Chronic Inflammation/ROS in Aging. Mol. Cell Biochem..

[67] Taylor E., Kim Y., Zhang K., Chau L., Nguyen B.C., Rayalam S., Wang X. (2022). Antiaging Mechanism of Natural Compounds: Effects on Autophagy and Oxidative Stress. Molecules.

[68] Guerrero-Navarro L., Jansen-Dürr P., Cavinato M. (2024). Synergistic Interplay of UV Radiation and Urban Particulate Matter Induces Impairment of Autophagy and Alters Cellular Fate in senescence-prone Human Dermal Fibroblasts. Aging Cell.

[69] Redza-Dutordoir M., Averill-Bates D.A. (2021). Interactions between Reactive Oxygen Species and Autophagy. Biochim. Biophys. Acta (BBA)—Mol. Cell Res..

[70] Cavagnino A., Azadiguian G., Breton L., Baraibar M., Black A.F. (2025). Modulating Skin Aging Molecular Targets and Longevity Drivers Through a Novel Natural Product: Rose-Derived Polydeoxyribonucleotide (Rose PDRN). Curr. Issues Mol. Biol..

[71] Gouin O., Cavagnino A., Azadiguian G., Jäger S., Comte G., Bendahmane M., Breton L., Baraibar M.A., Black A.F. (2025). Exploring Skin Longevity Pathways: Rosa Hybrid Extract-Mediated AMP-Activated Protein Kinase Activation, Antioxidant, and Autophagic Mechanisms in Human Keratinocytes. Cosmetics.

[72] Hseu Y.-C., Vudhya Gowrisankar Y., Wang L.-W., Zhang Y.-Z., Chen X.-Z., Huang P.-J., Yen H.-R., Yang H.-L. (2021). The in Vitro and in Vivo Depigmenting Activity of Pterostilbene through Induction of Autophagy in Melanocytes and Inhibition of UVA-Irradiated α-MSH in Keratinocytes via Nrf2-Mediated Antioxidant Pathways. Redox Biol..

[73] Kim C.-W., Alam M.B., Song B.-R., Lee C.H., Kim S.L., Lee S.-H. (2024). γ-Mangosteen, an Autophagy Enhancer, Prevents Skin-Aging via Activating KEAP1/NRF2 Signaling and Downregulating MAPKs/AP-1/NF-κB-Mediated MMPs. Phytomedicine.

[74] Zheng Q., Jin X., Nguyen T.T.M., Yi E.-J., Park S.-J., Yi G.-S., Yang S.-J., Yi T.-H. (2025). Autophagy-Enhancing Properties of *Hedyotis Diffusa* Extracts in HaCaT Keratinocytes: Potential as an Anti-Photoaging Cosmetic Ingredient. Molecules.

[75] Gu Y., Han J., Xue F., Xiao H., Chen L., Zhao Z., Zhang Y. (2022). 4,4′-Dimethoxychalcone Protects the Skin from AAPH-Induced Senescence and UVB-Induced Photoaging by Activating Autophagy. Food Funct..

[76] Gu Y., Xue F., Xiao H., Chen L., Zhang Y. (2022). Bamboo Leaf Flavonoids Suppress Oxidative Stress-Induced Senescence of HaCaT Cells and UVB-Induced Photoaging of Mice through P38 MAPK and Autophagy Signaling. Nutrients.

[77] Fernando P.D.S.M., Piao M.J., Kang K.A., Zhen A.X., Herath H.M.U.L., Kang H.K., Choi Y.H., Hyun J.W. (2022). Hesperidin Protects Human HaCaT Keratinocytes from Particulate Matter 2.5-Induced Apoptosis via the Inhibition of Oxidative Stress and Autophagy. Antioxidants.

[78] Woo J., Shin S., Ji H., Ryu D., Cho E., Kim Y., Kim J., Park D., Jung E. (2022). *Isatis Tinctoria* L. Leaf Extract Inhibits Replicative Senescence in Dermal Fibroblasts by Regulating mTOR-NF-κB-SASP Signaling. Nutrients.

[79] Wan K., Li J., Ma L., Chen T., Chen Y., Li Z., Zouboulis C.C., Wang G.-L., Wang J. (2025). Camellia Saponin Modulates Oleic Acid/Linoleic Acid-Induced Lipogenesis in Human Sebocytes through Lipophagy Activation. Int. J. Cosmet. Sci..

[80] Ono S., Kawasaki A., Tamura K., Minegishi Y., Mori T., Ota N. (2024). *Ruscus aculeatus* Extract Promotes RNase 7 Expression through ERK Activation Following Inhibition of Late-Phase Autophagy in Primary Human Keratinocytes. PLoS ONE.

[81] Deng X., Zhao F., Zhao D., Zhang Q., Zhu Y., Chen Q., Qiang L., Xie N., Ma J., Pan X. (2022). Oxymatrine Promotes Hypertrophic Scar Repair through Reduced Human Scar Fibroblast Viability, Collagen and Induced Apoptosis via Autophagy Inhibition. Int. Wound J..

[82] Huang L., Feng Z., Xiang J., Deng M., Zhou Z. (2024). Anti-Inflammatory Compounds from the Rhizome of *Acorus Calamus Var*. *Angustatus Besser* and Their Mechanism. Nat. Prod. Res..

[83] Martínez-Gutiérrez A., Fernández-Duran I., Marazuela-Duque A., Simonet N.G., Yousef I., Martínez-Rovira I., Martínez-Hoyos J., Vaquero A. (2021). Shikimic Acid Protects Skin Cells from UV-Induced Senescence through Activation of the NAD+-Dependent Deacetylase SIRT1. Aging.

[84] Lin F., Wang T., Ai J., Wang J., Huang C., Tian W., Lan T., Fu L., Chen X. (2025). Topical Application of Tea Leaf-Derived Nanovesicles Reduce Melanogenesis by Modulating the miR-828b/*MYB*4 Axis: Better Permeability and Therapeutic Efficacy than Conventional Tea Extracts. Mater. Today Bio.

[85] Okamoto S., Kakimaru S., Koreishi M., Sakamoto M., Nakamura Y., Ando H., Tsujino Y., Satoh A. (2025). Resveratrol, a Food-Derived Polyphenol, Promotes Melanosomal Degradation in Skin Fibroblasts through Coordinated Activation of Autophagy, Lysosomal, and Antioxidant Pathways. J. Funct. Foods.

[86] Martínez-Gutiérrez A., Sendros J., Noya T., González M.C. (2024). Apigenin and Phloretin Combination for Skin Aging and Hyperpigmentation Regulation. Cosmetics.

[87] Tang Q.-Q., Wang Z.-D., An X.-H., Zhou X.-Y., Zhang R.-Z., Zhan X., Zhang W., Zhou J. (2024). Apigenin Ameliorates H_2_O_2_-Induced Oxidative Damage in Melanocytes through Nuclear Factor-E2-Related Factor 2 (Nrf2) and Phosphatidylinositol 3-Kinase (PI3K)/Protein Kinase B (Akt)/Mammalian Target of Rapamycin (mTOR) Pathways and Reducing the Generation of Reactive Oxygen Species (ROS) in Zebrafish. Pharmaceuticals.

[88] Biscaro R.C., Mussi L., Sufi B., Padovani G., Camargo Junior F.B., Magalhães W.V., Di Stasi L.C. (2022). Modulation of Autophagy by an Innovative Phytocosmetic Preparation (Myrothamnus Flabelifolia and Coffea Arabica) in Human Fibroblasts and Its Effects in a Clinical Randomized Placebo-Controlled Trial. J. Cosmet. Dermatol..

[89] Duan Y., Xiang Y., Chu J., Lin X., He M., Zhang C., Sun S., Huang L. (2023). Handelin Reduces Ultraviolet A-Induced Photoaging by Inhibiting Reactive Oxygen Species Generation and Enhancing Autophagy. Tohoku J. Exp. Med..

[90] Choi W., Kim H.S., Park S.H., Kim D., Hong Y.D., Kim J.H., Cho J.Y. (2022). Syringaresinol Derived from *Panax Ginseng* Berry Attenuates Oxidative Stress-Induced Skin Aging via Autophagy. J. Ginseng Res..

[91] Yang K.E., Nam S.-B., Lee G.-E., Yang G., Lee M.-H., Bang G., Choi J.H., Cho Y.-Y., Lee C.-J. (2024). Induction of Autophagy by Extract from *Corydalis Heterocarpa* for Skin Anti-Aging. Mar. Drugs.

[92] Zhu S., Li X., Dang B., Wu F., Wang C., Lin C. (2022). *Lycium Barbarum* Polysaccharide Protects HaCaT Cells from PM2.5-Induced Apoptosis via Inhibiting Oxidative Stress, ER Stress and Autophagy. Redox Report..

[93] Cho Y.H. (2021). *Codonopsis Pilosula* Extract Protects Melanocytes against H_2_O_2_-Induced Oxidative Stress by Activating Autophagy. Cosmetics.

[94] Wang H., Zheng J., Cao S., Lv J. (2025). Research on the Mechanisms of Plant Bioactive Metabolites in Anti-Skin Aging and Future Development Prospects. Front. Pharmacol..

[95] Hartinger R., Fenzl F.Q., Nalewaja V.M., Djabali K. (2025). Argan Callus Extract Restores Skin Cells via AMPK-Dependent Regulation of Energy Metabolism, Autophagy, and Inflammatory Pathways. Antioxidants.

[96] Chen Q., Lin W., Tang Y., He F., Liang B., Chen J., Li H., Zhu H. (2025). Curcumin Targets YAP1 to Enhance Mitochondrial Function and Autophagy, Protecting against UVB-Induced Photodamage. Front. Immunol..

[97] Che L., Zhu C., Huang L., Xu H., Ma X., Luo X., He H., Zhang T., Wang N. (2023). Ginsenoside Rg2 Promotes the Proliferation and Stemness Maintenance of Porcine Mesenchymal Stem Cells through Autophagy Induction. Foods.

[98] Wang Y., Wang S., Wang R., Li S., Yuan Y. (2021). Neferine Exerts Antioxidant and Anti-Inflammatory Effects on Carbon Tetrachloride-Induced Liver Fibrosis by Inhibiting the MAPK and NF-κB/IκBα Pathways. Evid. Based Complement. Altern. Med..

[99] Bharathi Priya L., Huang C., Hu R., Balasubramanian B., Baskaran R. (2021). An Updated Review on Pharmacological Properties of Neferine—A Bisbenzylisoquinoline Alkaloid from *Nelumbo nucifera*. J. Food Biochem..

[100] Geyfman M., Chung R., Boissy R., Poloso N., Kadoya K., Maitra P., Mehta R. (2025). Lotus Sprout Extract Induces Selective Melanosomal Autophagy and Reduces Pigmentation. J. Cosmet. Dermatol..

[101] Ghorbel A., Wedel S., Kallel I., Cavinato M., Sakavitsi M.E., Fakhfakh J., Halabalaki M., Jansen-Dürr P., Allouche N. (2021). Extraction Yield Optimization of Oleaster (Olea Europaea Var. Sylvestris) Fruits Using Response Surface Methodology, LC/MS Profiling and Evaluation of Its Effects on Antioxidant Activity and Autophagy in HFF Cells. Food Meas..

[102] Yang H.-L., Lin C.-P., Vudhya Gowrisankar Y., Huang P.-J., Chang W.-L., Shrestha S., Hseu Y.-C. (2021). The Anti-Melanogenic Effects of Ellagic Acid through Induction of Autophagy in Melanocytes and Suppression of UVA-Activated α-MSH Pathways via Nrf2 Activation in Keratinocytes. Biochem. Pharmacol..

[103] Lin J., Lu Y.-Y., Shi H.-Y., Lin P. (2023). Chaga Medicinal Mushroom, *Inonotus Obliquus* (Agaricomycetes), Polysaccharides Alleviate Photoaging by Regulating Nrf2 Pathway and Autophagy. Int. J. Med. Mushrooms.

[104] Peng X., Ding C., Zhao Y., Hao M., Liu W., Yang M., Xiao F., Zheng Y. (2022). Poloxamer 407 and Hyaluronic Acid Thermosensitive Hydrogel-Encapsulated Ginsenoside Rg3 to Promote Skin Wound Healing. Front. Bioeng. Biotechnol..

[105] Alam M.B., Park N.H., Song B.-R., Lee S.-H. (2023). Antioxidant Potential-Rich Betel Leaves (*Piper Betle* L.) Exert Depigmenting Action by Triggering Autophagy and Downregulating MITF/Tyrosinase In Vitro and In Vivo. Antioxidants.

[106] Cho Y.H., Park J.E. (2024). Anti-Inflammatory and Autophagy Activation Effects of 7-Methylsulfonylheptyl Isothiocyanate Could Suppress Skin Aging: In Vitro Evidence. Antioxidants.

[107] Nguyen D.Q., Nguyen N.T.P., To T.T., Nguyen L.M.D., Pham T.K.V., Vu G.M., Lieu L.P. (2025). Double Nano-Emulsions for Stabilizing Vitamin C and Enhancing Antioxidant Capacity with Macadamia Oil and Tea Tree Essential Oil. Oil Crop Sci..

[108] El-Otmani N., Zeouk I., Zahidi A. (2024). Formulation of Biological Sunscreen from *Calendula Arvensis* Capitula Extracts: Antioxidant, Anti-Aging, Surface Tension, and UVB Protection Properties Assessed. Cosmetics.

[109] Zlabiene U., Baranauskaite J., Kopustinskiene D.M., Bernatoniene J. (2021). In Vitro and Clinical Safety Assessment of the Multiple W/O/W Emulsion Based on the Active Ingredients from *Rosmarinus officinalis* L., *Avena sativa* L. and *Linum usitatissimum* L.. Pharmaceutics.

[110] Nguyen M.M., Karboune S. (2023). Combinatorial Interactions of Essential Oils Enriched with Individual Polyphenols, Polyphenol Mixes, and Plant Extracts: Multi-Antioxidant Systems. Antioxidants.

[111] Taibi M., Elbouzidi A., Bentouhami N.E., Haddou M., Baraich A., Hammouti Y., Belbachir Y., Bellaouchi R., Mothana R.A., Hawwal M.F. (2025). Evaluation of the Dermatoprotective Properties of Clinopodium Nepeta and Thymus Vulgaris Essential Oils: Phytochemical Analysis, Anti-Elastase, Anti-Tyrosinase, Photoprotective Activities, and Antimicrobial Potential Against Dermatopathogenic Strains. Skin. Res. Technol..

[112] Oargă (Porumb) D.P., Cornea-Cipcigan M., Nemeș S.A., Cordea M.I. (2025). The Effectiveness of a Topical Rosehip Oil Treatment on Facial Skin Characteristics: A Pilot Study on Wrinkles, UV Spots Reduction, Erythema Mitigation, and Age-Related Signs. Cosmetics.

[113] Rahmi D., Yunilawati R., Jati B.N., Setiawati I., Riyanto A., Batubara I., Astuti R.I. (2021). Antiaging and Skin Irritation Potential of Four Main Indonesian Essential Oils. Cosmetics.

[114] Fayez S., Gamal El-Din M.I., Moghannem S.A., Azam F., El-Shazly M., Korinek M., Chen Y.-L., Hwang T.-L., Fahmy N.M. (2023). Eucalyptus-Derived Essential Oils Alleviate Microbes and Modulate Inflammation by Suppressing Superoxide and Elastase Release. Front. Pharmacol..

[115] Walendziak W., Villegas N.R., Douglas T.E.L., Kozlowska J. (2025). Phytochemical Studies of Plant Extracts Enclosed in Chitosan Microparticles and the Effect of Phytoformulations on Skin Condition. Eur. Polym. J..

[116] Nowak A., Zagórska-Dziok M., Perużyńska M., Cybulska K., Kucharska E., Ossowicz-Rupniewska P., Piotrowska K., Duchnik W., Kucharski Ł., Sulikowski T. (2022). Assessment of the Anti-Inflammatory, Antibacterial and Anti-Aging Properties and Possible Use on the Skin of Hydrogels Containing *Epilobium angustifolium* L. *Extracts*. Front. Pharmacol..

[117] Stefanowicz-Hajduk J., Nowak A., Hering A., Kucharski Ł., Graczyk P., Kowalczyk M., Sulikowski T., Muzykiewicz-Szymańska A. (2024). Antiaging Properties of Kalanchoe Blossfeldiana Ethanol Extract—Ex Vivo and In Vitro Studies. Molecules.

[118] Zhang Y., Sun B., Wang L., Shen W., Shen S., Cheng X., Liu X., Xia H. (2024). Curcumin-Loaded Liposomes in Gel Protect the Skin of Mice against Oxidative Stress from Photodamage Induced by UV Irradiation. Gels.

[119] Yazidi R., Hammami M., Ghadhoumi H., Ben Abdennebi A., Selmi S., Zayani K., Horchani-Naifer K., Bettaieb Rebey I., Saidani Tounsi M. (2025). Development and Optimization of a Quercetin-Loaded Chitosan Lactate Nanoparticle Hydrogel with Antioxidant and Antibacterial Properties for Topical Skin Applications. Cosmetics.

[120] Ding C., Liu Z., Zhao T., Sun S., Liu X., Zhang J., Ma L., Yang M. (2023). A Temperature-Sensitive Hydrogel Loaded with Taxifolin Promotes Skin Repair by Modulating MAPK-Mediated Autophagic Pathway. J. Mater. Sci..

[121] Gardiki V., Varvaresou A., Papageorgiou S., Protopapa E., Pavlou P., Rallis E., Papadopoulos A., Chaniotis D. (2025). A Randomized, Double-Blind, Placebo-Controlled Study Evaluating a Novel Skin Care Cream with Olea Europaea Stem Cell Extract Following Nd:YAG 1064 Nm Laser Epilation. Cosmetics.

[122] Kanai K., Biswas K.B., Hirasawa A., Futamura M., Tanaka K., Sakamoto K. (2025). Peony Root Extract Controls AGE–RAGE Interaction, Suppresses AGE Formation, and Reduces Skin Dullness. Cosmetics.

[123] Di Nicolantonio L., Trebbi G., Vargas Peregrina D., Rashad M., Censi R., Zara S., Di Martino P., Gigliobianco M.R. (2025). Development and Characterization of a New Cosmetic Formulation Enriched with Optimized Thanaka Bark Extract (*H. Crenulata*). J. Drug Deliv. Sci. Technol..

[124] Moneo-Sánchez M., de Pablo N., Arana-Pascual L., Beitia I., Benito-Cid S., Pérez-González R. (2024). Multifunctional, Novel Formulation for Repairing Photoaged and Sun-Damaged Skin: Insights from In Vitro, Ex Vivo, and In Vivo Studies. Cosmetics.

[125] Wang G., Fei W., Zhi L., Bai X., You B. (2025). Fermented Tea Leave Extract against Oxidative Stress and Ageing of Skin in Vitro and in Vivo. Int. J. Cosmet. Sci..

[126] Maya I., Sriwidodo S., Mita S.R., Kusumawulan C.K., Putriana N.A., Amalia E., Aulia R.N., Sofyan H.N., Dzulfannazhir F., Nugraha M.H. (2024). Formulation and Evaluation of Facial Serum Containing Sacha Inchi Oil (*Plukenetia volubilis* L.) from Indonesia as an Anti-Aging: Stability, In Vitro, and Skin Irritation Assessments. Cosmetics.

[127] Mungmai L., Wongwad E., Tanamatayarat P., Rungsang T., Vivattanaseth P., Aunsri N., Preedalikit W. (2025). Stability, Bioactivity, and Skin Penetration of Prunus Leaf Extracts in Cream Formulations: A Clinical Study on Skin Irritation. Cosmetics.

[128] Oh S., Jeong J., Kim M., Jin X., Zheng S., Kim Y.-M., Yi T.-H. (2024). A Study of Anti-Wrinkle Functions and Improvement of Cream with Phaseolus Angularis. Int. J. Cosmet. Sci..

[129] Infante V.H.P., Campos P.M.B.G.M., Gaspar L.R., Darvin M.E., Schleusener J., Rangel K.C., Meinke M.C., Lademann J. (2022). Safety and Efficacy of Combined Essential Oils for the Skin Barrier Properties: In Vitro, Ex Vivo and Clinical Studies. Int. J. Cosmet. Sci..

